# High Arctic seawater and coastal soil microbiome co-occurrence and composition structure and their potential hydrocarbon biodegradation

**DOI:** 10.1093/ismeco/ycae100

**Published:** 2024-07-16

**Authors:** Nastasia J Freyria, Esteban Góngora, Charles W Greer, Lyle G Whyte

**Affiliations:** Department of Natural Resource Sciences, Faculty of Agricultural and Environmental Sciences, McGill University, 21111 Lakeshore Road, Macdonald Stewart Building, Room MS3-053, Sainte-Anne-de-Bellevue, QC H9X 3V9, Canada; Department of Natural Resource Sciences, Faculty of Agricultural and Environmental Sciences, McGill University, 21111 Lakeshore Road, Macdonald Stewart Building, Room MS3-053, Sainte-Anne-de-Bellevue, QC H9X 3V9, Canada; Department of Natural Resource Sciences, Faculty of Agricultural and Environmental Sciences, McGill University, 21111 Lakeshore Road, Macdonald Stewart Building, Room MS3-053, Sainte-Anne-de-Bellevue, QC H9X 3V9, Canada; Energy, Mining and Environment, Research Centre, National Research Council Canada, 6100 Royalmount Ave., Montreal, QC, H4P 2R2, Canada; Department of Natural Resource Sciences, Faculty of Agricultural and Environmental Sciences, McGill University, 21111 Lakeshore Road, Macdonald Stewart Building, Room MS3-053, Sainte-Anne-de-Bellevue, QC H9X 3V9, Canada

**Keywords:** polar microbial community, bacteria, microbial eukaryotes, soil sediment, surface seawater, hydrocarbon degradation, Northwest Passage, Nanopore MinION

## Abstract

The accelerated decline in Arctic sea-ice cover and duration is enabling the opening of Arctic marine passages and improving access to natural resources. The increasing accessibility to navigation and resource exploration and production brings risks of accidental hydrocarbon releases into Arctic waters, posing a major threat to Arctic marine ecosystems where oil may persist for many years, especially in beach sediment. The composition and response of the microbial community to oil contamination on Arctic beaches remain poorly understood. To address this, we analyzed microbial community structure and identified hydrocarbon degradation genes among the Northwest Passage intertidal beach sediments and shoreline seawater from five high Arctic beaches. Our results from 16S/18S rRNA genes, long-read metagenomes, and metagenome-assembled genomes reveal the composition and metabolic capabilities of the hydrocarbon microbial degrader community, as well as tight cross-habitat and cross-kingdom interactions dominated by lineages that are common and often dominant in the polar coastal habitat, but distinct from petroleum hydrocarbon-contaminated sites. In the polar beach sediment habitats, *Granulosicoccus* sp. and *Cyclocasticus* sp. were major potential hydrocarbon-degraders, and our metagenomes revealed a small proportion of microalgae and algal viruses possessing key hydrocarbon biodegradative genes. This research demonstrates that Arctic beach sediment and marine microbial communities possess the ability for hydrocarbon natural attenuation. The findings provide new insights into the viral and microalgal communities possessing hydrocarbon degradation genes and might represent an important contribution to the removal of hydrocarbons under harsh environmental conditions in a pristine, cold, and oil-free environment that is threatened by oil spills.

## Introduction

**Figure 1:**
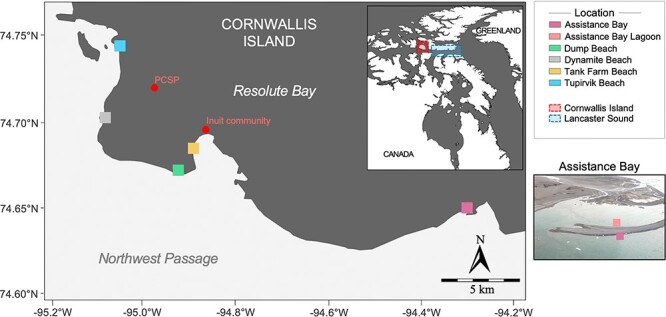
Costal map of Resolute Bay. Map of Resolute Bay on Cornwallis Island, Nunavut, Canada. The photography shows the location site of Assistance Bay and Assistance Bay lagoon. The photography was taken in July 2022 by N.J.F.

In the wake of ongoing climate change, the Arctic Ocean is highly impacted by the spring freshet in surface salinity, due to the melting of snow and ice, and the reduction in sea-ice thickness and concentration. According to models, the Arctic summer will be ice-free in this century [1]. The Northwest Passage (NWP) is a sea route that connects the Pacific Ocean and the North Atlantic Ocean through the Canadian Arctic Archipelago (Fig. 1). Lancaster Sound in the NWP serves as a major migratory route for marine ecosystems, supporting numerous marine and terrestrial species [2, 3]. While there is increasing interest in utilizing the NWP as a less-costly transportation route [4, 5], it is essential to consider the potential impact on the fragile Arctic marine ecosystem. There is a growing risk of contamination in polar biomes that have limited exposure to human industrial activities. This is particularly true in remote regions like the Canadian high Arctic, which lacks the necessary infrastructure to quickly respond to an oil spill. Although many oil cleanup strategies have been effective in various environments, the Arctic’s unique conditions make many methods inapplicable or expensive [6]. Severe weather conditions in the high Arctic region, such as extreme low temperatures, strong winds, and sea-ice movement [7], pose a safety risk to responding properly to an oil spill and effectively deploying cleanup strategies due to the costs of operating in such remote locations. To mitigate the risk of heavy fuel oil (HFO) contamination in Arctic waters, a number of Canadian organizations, including Indigenous and Inuit groups and the International Convention for the Prevention of Pollution from Ships (MARPOL), have proposed a ban on the use of HFO by ships navigating the Arctic [8, 9]. However, ships using other types of fuel may still pose a threat to Arctic marine and beach environments. Recently, the largest increases in shipping and oil spill potential are related to the exploration of natural resources, particularly those associated with mining sites, such as Nanisivik on Baffin Island [10].

Petroleum hydrocarbons are the main source of pollution in polar ecosystems [11, 12] and can persist in subtidal sediments and beach environments, fundamentally affecting the environmental health of the marine ecosystem and the local Inuit community [13]. During oil spills, hydrocarbon compounds can become trapped in coastal intertidal zones and may be pushed into supratidal zones [14]. This was observed during the “Exxon Valdez” oil spill in southern Alaska [15] and the “Deepwater Horizon” explosion in the Gulf of Mexico [16], which caused devastating effects on coastal areas. Therefore, it is necessary to conduct systematic studies and ongoing monitoring of biodegradation processes in the NWP, especially in coastal intertidal environments. The natural attenuation of petroleum hydrocarbons in the marine environment is a well-known phenomenon. Extensive research has been conducted on this topic [17–21], and it is widely accepted that contaminants in environmental systems are transformed by natural physical, chemical, and/or biological processes. Biodegradation of petroleum can occur at sub-zero temperatures in Arctic seawater mesocosms (−1°C) and Arctic sea-ice microcosms (−1.7°C) [22, 23]. However, much less is known about the natural degradation of hydrocarbons in Arctic coastal intertidal sediments. A follow-up study of the BIOS control oil spill experiment on northern Baffin Island [24] demonstrated that natural attenuation processes were insufficient to completely remediate hydrocarbons when petroleum was left to degrade under natural conditions in NWP beach sediments, even after 40 years.

Evidence of natural attenuation of hydrocarbons in Arctic environments using high-throughput sequencing approaches is limited, particularly in the Canadian context. An effective multi-omics approach is necessary for a comprehensive assessment regarding the capabilities of endemic microorganisms to degrade hydrocarbon compounds. The response of indigenous microbial communities to an oil spill in the harsh Arctic environment, the effectiveness of bioremediation treatments, and the prediction of specific natural rates of hydrocarbon degradation remain uncertain and difficult to ascertain. Our perspective focuses on the applicability of biodegradation driven by endemic hydrocarbon-degrading microorganisms. Therefore, we collected polar intertidal beach sediments and shoreline surface seawater from five Canadian high Arctic NWP beaches during the summers of 2018–2022. To characterize the composition and abundance of the endemic microbial communities and identify cross-habitat and cross-kingdom correlations among the coastal microbial consortia, we performed 16S/18S rRNA gene amplicon sequencing. We screened MinION-derived metagenomes and metagenome-assembled genomes (MAGs) for the presence of key hydrocarbon biodegradation (HB) genes among these lineages. The results will be essential to develop an effective bioremediation strategy for contaminated Arctic shorelines.

## Materials and methods

### Site description, sample collection, and environmental variables measurements

Five NWP beaches were sampled from areas adjacent to Resolute Bay (RB), Cornwallis Island, Nunavut, in the Canadian high Arctic Archipelago (Fig. 1). RB is in a region that remains covered in ice for at least 10 months of the year and has an annual air temperature of −15.7°C, with only 3 months above 0°C. A total of 41 samples were gathered from the intertidal zone of the Resolute coastline, comprising 19 sediment samples and 22 surface seawater samples during the summers of 2018, 2019, 2021, and 2022 (Table S1; see Supplementary Materials). We measured *in-situ* seawater temperature, salinity, and pH using a YSI ProQuatro Multiparameter Instrument (Xylem Inc., Yellow Springs, OH, USA). Dissolved oxygen (DO) was measured *in situ* with a PyroScience Picco-2 oxygen meter (Aachen, Germany). Nitrate and phosphate concentrations were measured using CHEMetrics Inc. test kits (K-8503 and K-6903) with the V-2000 CHEMtrics photometer.

### Seawater microbial cell concentrations by flow cytometry

The abundance of marine microbial phytoplankton and marine bacteria from shoreline surface seawater was assessed using an Accuri C6 flow cytometer (BD Biosciences, Franklin Lakes, NJ, USA; Table S2) as previously described [25]. Cell enumeration and data acquisition methods were followed as previously described [26, 27]. Eukaryotic pico- and nanophytoplankton cells were differentiated using chlorophyll red fluorescence and side-scattered light at 670 nm [27].

### DNA and RNA extraction, library preparation, and sequencing

DNA was extracted from 0.5 g of beach sediment, and both DNA and RNA were extracted from surface seawater samples using both 0.2 μm polycarbonate filters (AMD Manufacturing Inc., Mississauga, ON, Canada) and Sterivex filtration units (Sigma-Aldrich, Oakville, ON, Canada) as previously described [25]. The resulting DNA and complementary DNA (cDNA; see Supplementary Materials) from each sample were concentrated, barcoded, pooled, and sequenced in-house on a MiSeq platform (Illumina, San Diego, CA, USA) for 16S and 18S rRNA gene sequencing, and on a MinION Mk1c (MC-110217, Oxford Nanopore Technologies, Oxford, UK) device using R9.4 FLO-MIN106 flow cells (Oxford Nanopore Technologies) for long-read metagenomes.

### Sequence data processing

To generate amplicon sequence variants (ASVs), all 16S/18S rRNA gene sequences were processed using the R package *dada2* [28]. Taxonomy was assigned using the Silva Reference Database [29] and the Protist Ribosomal Reference database [30]. The 16S/18S rRNA gene AVSs matrix was filtered and rarefied as previously described [25]. All metagenomes MinION sequences were assembled using Flye [31]. MetaErg [32] was used to annotate the assembled metagenome contigs. Carbohydrate-active enzymes were predicted using dbCAN based on the CAZy database [33]. The CANT-HYD [34] approach was utilized to detect the presence of HB genes. Contigs were binned to reconstruct MAGs (Table S3), using MetBAT2 [35], MaxBin2 [36], and CONCOCT [37]. Only contigs longer than 1500 bp were retained. The completeness and contamination level of all bins were assessed using CheckM [38], and only bins with a contamination level below 15% and a completeness >50% were kept [39]. The replication of bins was verified using dRep [40], resulting in a total of 63 filtered medium-quality MAGs (Table S4). No high-quality MAGs (completeness >90% and contamination <5%) were obtained with our data. Bins were classified using the Genome Taxonomy Database Toolkit [41] and annotated with MetaErg.

### Statistical analyses

Beta diversity was analyzed using Constrained Correspondence Analysis (CCA) in coordination with the R package vegan. A permutational multivariate analysis of variance (PERMANOVA) was employed using the R package vegan to ascertain the noteworthy effects of environmental factors on the combined community (Table S5). Spearman’s rank correlation analysis was conducted using the R package corrgram. CoNet plugin [42] in Cytoscape was used to construct co-occurrence networks solely from the top 500 most abundant ASVs.

## Results

### Polar coastal microbiome structure and environmental drivers

**Figure 2:**
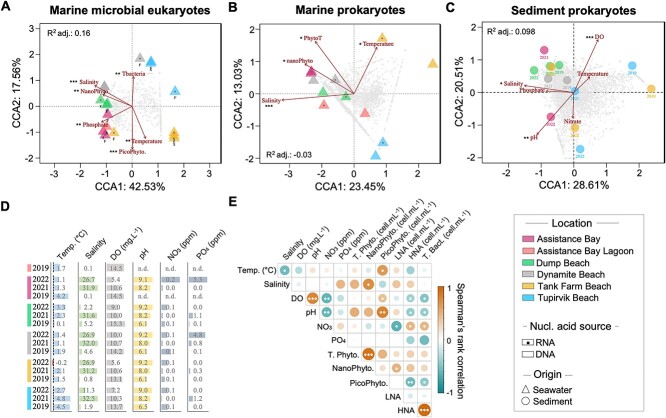
Clustering of microbial eukaryote and prokaryote communities with environmental variables. Constrained Correspondence Analysis (CCA) ordinations of the (A) 18S rRNA gene and of the (B) and (C) 16S rRNA gene sequences from shoreline surface seawater samples. Only samples from July 2022 are computed on each CCA with environmental parameters. Arrows represent environmental parameters: dissolved oxygen (DO, mg L^−1^); concentration in cells ml^−1^ of nanophytoplankton (NanoPhyto, <3 μm), of picophytoplankton (PicoPhyto, >3 μm), and of total bacteria (Tbacteria); phosphate concentration (ppm); and seawater temperature (°C). Letter “F” indicates the use of Polycarbonate filter and letter “S” indicates the use of Sterivex filter to filter seawater from July 2022 (see Supplementary Methods). Symbol of a “+” represents species. (D) Environmental measurement values for each beach during summers of 2019, 2021 and 2022. (E) Spearman’s rank correlation (*r*) matrix between environmental variables in July 2022. Spearman correlation coefficient values are represented by the size of the circles (larger for high coefficients; smaller for low coefficients). Empty squares indicate no correlation. Asterisks within circles represent the significance level of *P*-value from Spearman’s Rho (*P*-value <.05^*^, *P*-value <.01^*^^*^, *P*-value <.001^*^^*^^*^). A complete list of environmental values is in Table S1.

Water temperature varied considerably depending on the year of sampling (Fig. 2A; Table S1). In July 2019, the water temperature reached 4.5°C (Tupirvik Beach), while it was only 0.1°C at the Dump Beach in the same year and month of sampling. The lowest seawater temperature recorded was −0.2°C in July 2022 (Dump Beach). Seawater salinity ranged from 0.01% (Assistance Bay) to 0.51% (Dump Beach) in July 2019. This was consistent with the high concentration of sea-ice that summer. In August 2021, the salinity ranged from 3.11% (Dynamite Beach) to 3.26% (Tupirvik Beach), which is consistent with the low amounts of sea-ice observed that summer. Nutrient concentrations remained consistently low throughout all summers (nitrate: 0.067 ± 0.03 ppm; phosphate: 0.908 ± 0.46 ppm), with the highest concentration recorded at Assistance Bay in July 2022 (nitrate: 0.24 ppm; phosphate: 5.25 ppm; Table S1).

The analysis of Spearman’s rank correlation for the 2022 environmental variables revealed that the concentrations of total bacterial cells and high nucleic acid (HNA) cells exhibited a similar correlation pattern (Fig. 2B). They were significantly and positively correlated with nitrate concentration (*P*-value <.05), but negatively correlated with pH, DO, and picophytoplankton (<3 μm) cells (*P*-value <.05). The abundance of prokaryotic and nanophytoplankton (>3 μm) cells in the seawater samples was found to be higher at the Dynamite Beach than at the other beaches (Table S2).

CCA ordination analyses indicated that temperature and salinity have a significant impact on the variation in the composition of the marine microbial eukaryote (temperature: *P*-value <.01^*^^*^; salinity: *P*-value <.001^*^^*^^*^), marine prokaryote (temperature: *P*-value <.05^*^; salinity: *P*-value <.001^*^^*^^*^), and sediment prokaryote (salinity: *P*-value .05^*^) communities across all beaches (Fig. 2C–E). To confirm environmental influences on the microbial community, PerMANOVA test analyses indicated that salinity had the strongest significant correlation with the eukaryote community (*R^2^* = 0.22, *F* = 11.43, *P*-value <.001^*^^*^^*^, adjusted *P*-value <.004^*^^*^) and with the marine prokaryote community (*R*^2^ = 0.26, *F* = 2.76, *P*-value = .042^*^, adjusted *P*-value = .21; Table S5).

### Polar coastal core microbiome composition and distribution

**Figure 3:**
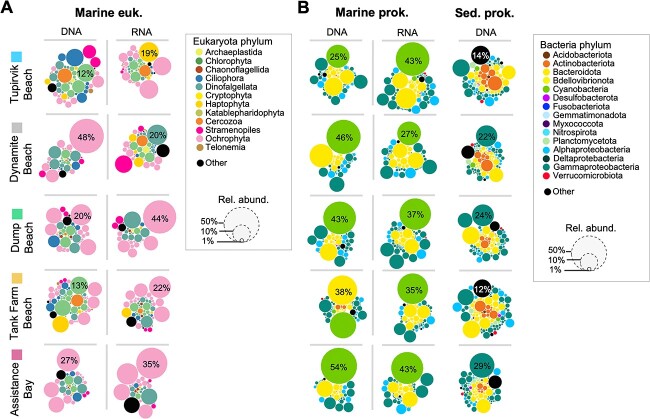
Global coastal microbial composition of sediment and seawater samples. (A) Circle plots indicate the taxonomical composition of both (A) 18S and (B) 16S rRNA genes (DNA dataset) and its transcriptional products (RNA dataset) of shoreline marine eukaryotes (euk.), marine prokaryotes (prok.), and beach sediment (Sed.) prokaryote communities. Only ASVs of DNA and RNA datasets are represented for marine samples. Size of each circle represents the relative abundance (Rel. abund.) of each genus in the overall (A) marine eukaryotes community and (B) marine and sediment prokaryotes communities. The color of each circle represents a genus among the most dominant phylum level. The black color represents other and unknown taxa. Only the proportion of abundance of the most dominant taxa are indicated in percentage.

To gain insight into the ecology of the region’s dominant taxa, the microbial abundance and diversity were identified in five Arctic NWP beaches (sediment and seawater) using 16S/18S rRNA gene amplicon sequencing and MinION metagenomic sequencing (Figs 3 and S1–S5). In the marine prokaryote community, *Cyanobacteria* was the most dominant phylum in both the 16S rRNA gene (DNA dataset) and its transcriptional products (RNA dataset), representing the potential active cells (Fig. 3). The proportion of *Cyanobacteria* ranged from 25% at the Tupirvik Beach to more than half of the total marine community (54%) at Assistance Bay in the DNA dataset. Only at the Tank Farm Beach did *Bacteroidota* dominate the community (38%). In contrast, the DNA community of sediment prokaryotes revealed that the class *Gammaproteobacteria* was the most abundant at three beaches: Dynamite (22%), Dump (24%), and Assistance Bay (29%). Overall, the diversity of the prokaryotic community in the sediments exceeded that of the marine prokaryotic community, which was mainly composed of Proteobacteria, *Bacteroidota*, and Actinobacteriota. The phylum *Ochrophyta* (*Stramenopiles*) was the most dominant at all beaches, ranging from 22% (Tank Farm) to 44% (Dynamite) of the total marine microbial eukaryotic community of both DNA and RNA datasets. In contrast, *Cryptophyta* was dominating at the Tupirvik Beach (19%, RNA dataset), and *Chlorophyta* dominated the community at two beaches (Tupirvik: 12%; Tank Farm: 13%, DNA dataset). Furthermore, we identified the top 50 ASVs in sediment and seawater microbial communities (Fig. S1). Several ASVs classified as *Granulosicoccus* (*Gammaproteobacteria*) were predominantly present in sediments from July 2019 (Dump Beach), while the genus was absent from the top 50 AVSs in the marine bacterial community (Fig. S1A and B). In the seawater eukaryotic community, *Stramenopiles* were the most retrieved phylum among the top 50 ASVs (Fig. S1C), with 26 ASVs classified as *Bacillariophyta*.

Nanopore metagenomic taxonomic assignment revealed differences in the prokaryotic community of seawater samples compared to amplicon data, where *Cyanobacteria* reads represented a small proportion and the community was dominated by mostly *Flavobacteriaceae* (Fig. S2). Within the class *Gammaproteobacteria*, *Granulosicoccaceae* dominated almost all beach sediment samples, while *Nitrincolaceae* dominated seawater samples. *Alphaproteobacteria* were present in lower proportions in all sediment samples. Additionally, in microbial eukaryotes, the community was dominated by *Chlorophyta*, specifically the *Mamiellaceae* family (Fig. S3). The archaeal community in metagenomes was consistent across sampling sites, where three phyla—*Halobacterota*, *Euryarchaeota*, and *Thermoplasmatota*—dominated the communities (Fig. S4). Viral DNA was found in a larger proportion (i.e. Dump Beach: 9.6%) in seawater than in sediment samples (Fig. S5).

### Co-occurrence pattern of the polar coastal microbiome

**Figure 4:**
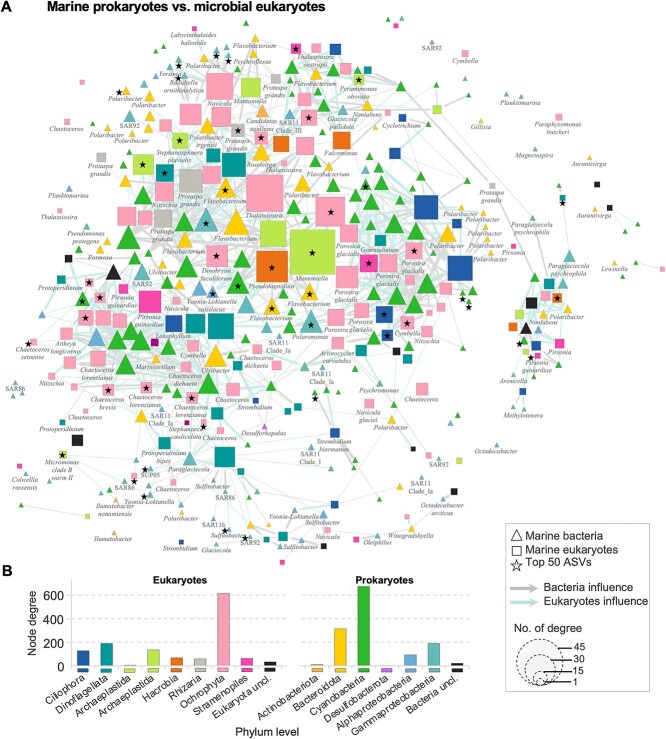
Pattern of co-occurrence between cross-kingdom and cross-habitat of the coastal seawater of the top 500 ASVs of 16S and 18S communities. Only mutual presence of ASVs is shown (mutual exclusions are removed from the network). The total number of nodes for (A) is 327, with 711 total number of edges and. Colors correspond to the phylum level. The size of the node is proportional to the number of connections. The edges represent positive and negative correlations between two nodes. Each star represents the presence of the top 50 ASVs from Fig. S1. Colors of each arrow represent the direction of correlation. (B) Gray arrow start from a marine bacterial node to a marine eukaryote node. Bar plots represent the number of node connection (node degree) for each phylum level.

**Figure 5:**
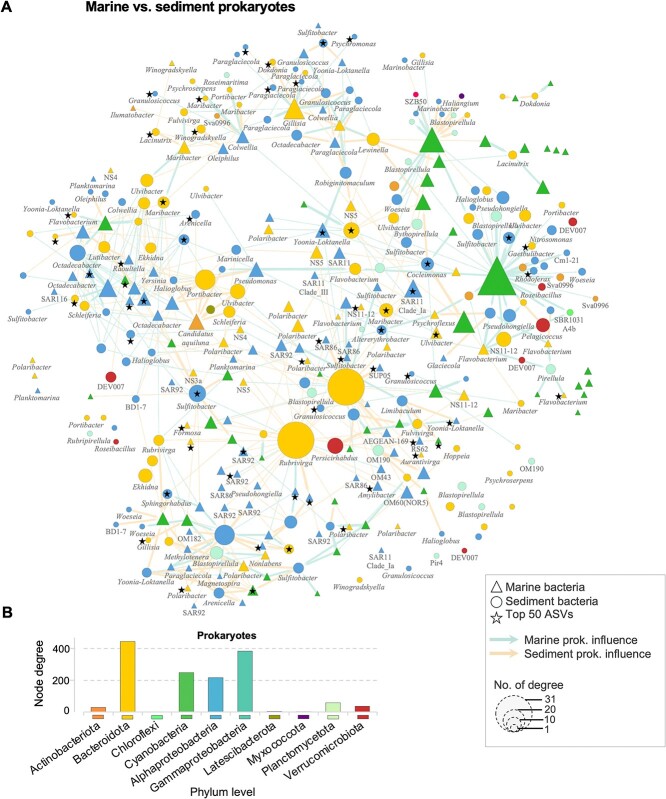
Pattern of co-occurrence between cross-kingdom and cross-habitat of the coastal sediment and seawater of the top 500 ASVs of 16S communities. Only mutual presence of ASVs is shown (mutual exclusions are removed from the network). The total number of nodes for is 374, with 1308 total number of edges. The size of the node is proportional to the number of connections. The edges represent positive and negative correlations between two nodes. Each star represents the presence of the top 50 ASVs from Fig. S1. Each arrow represent the direction of correlation. Bar plots represent the number of node connection (node degree) for each phylum level.

We investigated the co-occurrence patterns to predict significant associations between bacterial and microbial eukaryotic communities in beach sediment and shoreline seawater (Figs 4 and 5). The degree of an individual ASV indicates its level of interaction with other ASVs. The analyses identified hub species (high-degree connections) and potential species interactions in niche sharing, including cross-kingdom and cross-environment interactions. The co-occurrence network of marine bacterial and microbial eukaryotes showed that *Stramenopiles* were primarily associated with *Cyanobacteria* and several members of *Flavobacteriales*, including the genera *Polaribacter*, *Flavobacterium*, and *Nonlabens* (Fig. 4). Furthermore, the co-occurrence of the bacterial community in both marine and sediment environments showed a tight correlation among *Cyanobacteria* and members of *Flavobacteriales*, specifically within the genera *Rubrivirga*, *Portibacter*, *Maribacter*, *Polaribacter*, *Gillisia*, *Portibacter*, and *Ulvibacter* (Fig. 5). Additionally, *Cyanobacteria* was also connected with members of *Proteobacteria*, including the genera *Granulosicoccus*, *Cycloclasticus*, *Nitrosomonas*, *Woeseia*, *Sulfitobacter*, *Marinobacter*, *Rhodoferax*, *Octadecabacter*, SAR86, and SAR92.

### Hydrocarbon metabolic potentials of the high Arctic coastal microbiome

The distribution of the alkane and aromatic compound degradation pathways in the polar coastal (both sediment and seawater) environment was measured using a selection of 52 unique HB genes for these pathways (Tables S6 and S7). Only taxa possessing at least one read among the 52 unique HB genes were kept. Among this selected community, *Granulosicoccus antarcticus* dominated, ranging from 6.5% to 28.7%, except at the Tank Farm Beach, where *Cycloclasticus* sp. (6.5%) was the most dominant (Fig. S6). Among the class *Alphaproteobacteria*, *Roseovarius aesturii* was the most prevalent potential hydrocarbon-degrader at three beaches (Dynamite, Dump, and Assistance Bay), while *Ascidiaceihabitans* sp. was more abundant at the Tupirvik Beach, and *Ruegeria* sp. at the Tank Farm Beach (Table S8).

**Figure 6:**
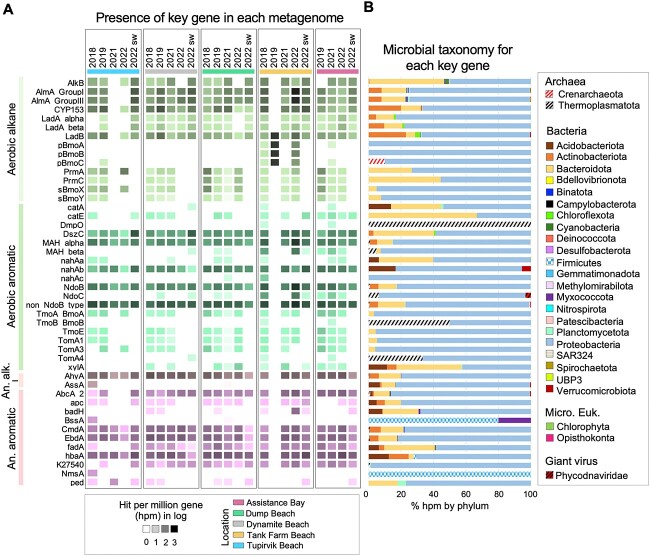
Distribution of hydrocarbon-degraders identified in long-read metagenomes across the coastal Canadian high Arctic. (A) Heatmap indicates the presence of each hydrocarbon degradation gene in all samples. Counts per sample were normalized to log hits per million (hpm) coding genes. Seawater samples are highlighted for each beach by year “2022 SW”, and year 2018 to 2022 represent sediment samples. (B) Bar plots represent the total number of unique species calculated in percentage of hpm with a known classification for each location. A corresponding table for each hydrocarbon biodegradative gene and the taxonomy affiliation is in Tables S5 and S6, respectively.

The most abundant HB genes found in sediment metagenomes included the putative beta subunit of polycyclic aromatic hydrocarbon (PAH) dioxygenases (non-*ndoB* type), the 4-hydroxybenzoate-CoA ligase (*hbaA*; K04105), the alkane C2 methylene hydroxylase (*ahyA*), the cytochrome P450 (CYP153), and the long-chain alkane oxidizing enzyme (*alma* group I; Fig. 6A). Several HB genes for the aliphatic degradation pathway were absent in seawater metagenomes, including particulate butane monooxygenase (*pBmoABC*; K10944, K10945, and K10946), propane 2-monooxygenase (*prmA* and *prmC*; K18223 and K18224), and soluble butane monooxygenase (*sBmoXY*; K16157 and K16158). Similarly, several HB genes for PAH degradation pathways were absent in seawater metagenomes, including toluene-2-monooxygenase (*tmoA1*, *tmoA3*, and *tmoE*; K16243, K16242, and K15764), and toluene-benzene monooxygenase (*tmoA_BmoA* and *tmoB_BmoB*; K15760 and K15761). Additionally, our metagenomes revealed the presence of a microbial alga, *Ostreococcus tauri* (Mamiellophyceae) encoding *nahAa* (K14581), a naphthalene 1,2-dioxygenase ferredoxin reductase component, which was exclusively found at the Dynamite Beach (Fig. 6B, Table S7). Microalgae represented only a small fraction of the total metagenome reads (from 0% to 2.3%; Fig. S5). Algal viruses assigned to *Prymnesiovirus* (*Phycodnaviridae*) were detected in a small proportion (from 0% to 9.6%; Fig. S5) in our metagenomes. We identified the presence of several HB genes classified ad *Prymnesiovirus* in the metagenomes of Dynamite and Tupirvik beaches, including *alma* groups I and III, and *ndoc* (K14579), a naphthalene 1,2-dioxygenase beta subunit (Fig. 6B, Table S7).

### KEGG metabolic pathways detected in MAGs

Using MinION-derived metagenomes, we reconstructed 63 bacterial MAGs of medium-quality (Table S5) that displayed diverse hydrocarbon metabolic potentials and reflected their taxonomic affiliation and the environment they were sourced from, either seawater or beach sediment. Fifteen sediment MAGs and sixteen seawater MAGs were primarily affiliated with taxa within the phylum *Bacteroidota*, with the majority being *Flavobacteriaceae*, including the genera *Maribacter* (four sediment MAGs) and *Patiriisocius* (two seawater MAGs; Fig. S7). The sediment MAGs were commonly populated by *Gammaproteobacteria*, while *Alphaproteobacteria* were prevalent in seawater MAGs. Additionally, two other sediment MAGs affiliated with *Cycloclasticus* sp. were identified. Most MAGs showed potential for carbohydrate and energy metabolism, including carbon fixation, as well as nitrogen and sulfur metabolism. An incomplete pathway of methane metabolism (ko00680), including incomplete methane oxidation (KEGG module M00174), was observed in numerous sediment and seawater MAGs, primarily within the family *Porticoccaceae* (*Gammaproteobacteria*; Fig. S7, Table S9). Various autotrophic carbon fixation pathways, including the Calvin–Benson cycle, Arnon–Buchanan cycle, Wood–Ljungdahl cycle, 3-hydroxypropionate bicycle, and dicarboxylate-hydroxybutyrate cycle, were present in some of the MAGs (Fig. S7, Table S9). All MAGs contained carbohydrate-active enzymes (CAZYME), with a total of 129 different CAZYMEs, indicating a strong potential for degrading complex carbon substrates. Sediment MAGs exhibited a broader distribution of pathways for aromatic degradation than seawater MAGs, including benzoate, toluene, and xylene degradation.

### Metabolic prediction of hydrocarbon degradation presence in MAGs

**Figure 7:**
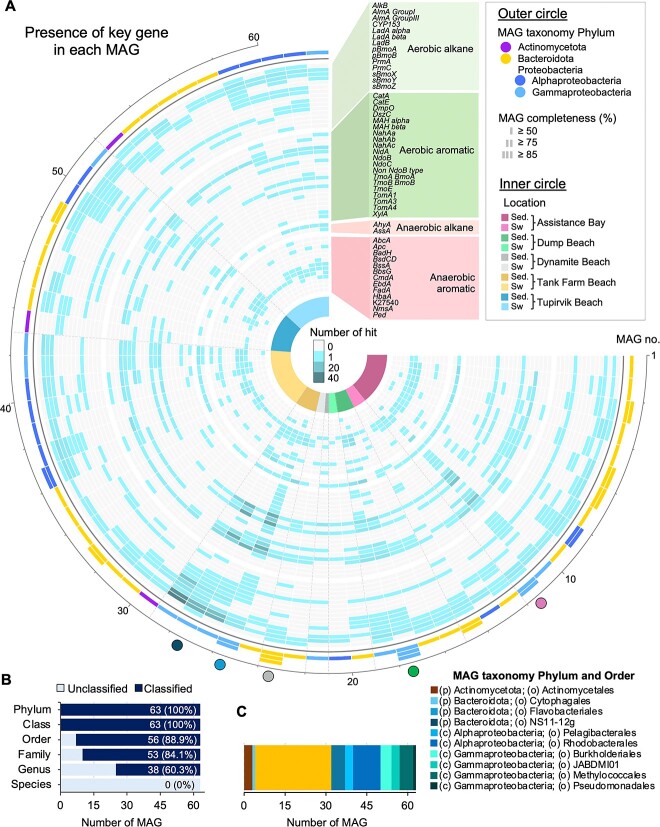
Hydrocarbon biodegradative (HB) genes found in 63 metagenome-assembled genomes (MAGs) from nanopore MinION sequencing. (A) Circular heatmap of HB genes present in each MAG. Colors in the outer circle represent the taxonomic affiliations of each MAG (phylum and class levels). Colors in the inner circle represent each beach site with lighter colors for seawater samples and darker colors for sediment samples: AB—Assistance Bay, DP—Dump Beach, DY—Dynamite Beach, TK—Tank Farm, and TU—Tupirvik Beach. (B) Bar plot represents the total number of MAGs and the number of known and unknown classifications. (C) Bar plot representing the taxonomy at the lowest level for each MAG. The five selected MAGs (see Fig. 8) are indicated by a colored circle outside the outer circle of the circular heatmap. A corresponding table for each HB gene present in each MAG is in Table S9.

The metabolic capabilities of each MAG were investigated, and HB genes were identified in a diverse range of taxonomic groups of MAGs (Fig. 7, Table S10). The gene *alkB* (K00496) was found in several bacterial phyla, including *Proteobacteria* with the genera *Ascidiaceihabitans*, *Amylobacter*, and *Yoonia*; *Actinomycetota* with the genus *Aquilina*; and *Bacteroidota* genera (*Aureibaculum*, *Maribacter*, *Patiriisocius*, and *Urechidicola*)*.* Furthermore, the HB genes *amla* group I, *ahyA*, an alkane C2 methylene hydroxylase, and *ladA* (K20938), a long-chain alkane monooxygenase within the alkane degradation pathway, were predominantly present. MAGs affiliated with the order *Pseudomonadales* had the highest number of gene counts for several HB genes, including *alma* group I, *ndoB* (K14579), and *edbA* (K10700), an ethylbenzene dehydrogenase. MAG-26 (75.43% completeness, 1.65% contamination; Tables S9 and S10) from the Tank Farm Beach, was assigned to *Cyclocasticus* sp., and possessed almost all HB genes and degradation pathways, including a high number of gene counts for several aromatic-degradative HB genes, including *dszC* (K22219), a dibenzothiophene desulfurization enzyme C, MAH alpha and beta, *ndoB*, *ndoC*, and non-*ndoB* type (Fig. 7A). Only 60% of all reconstructed MAGs were classified to the genus level, and almost 40% of the MAGs had no known genus or species assigned (Fig. 7B and C, Table S10).

**Figure 8:**
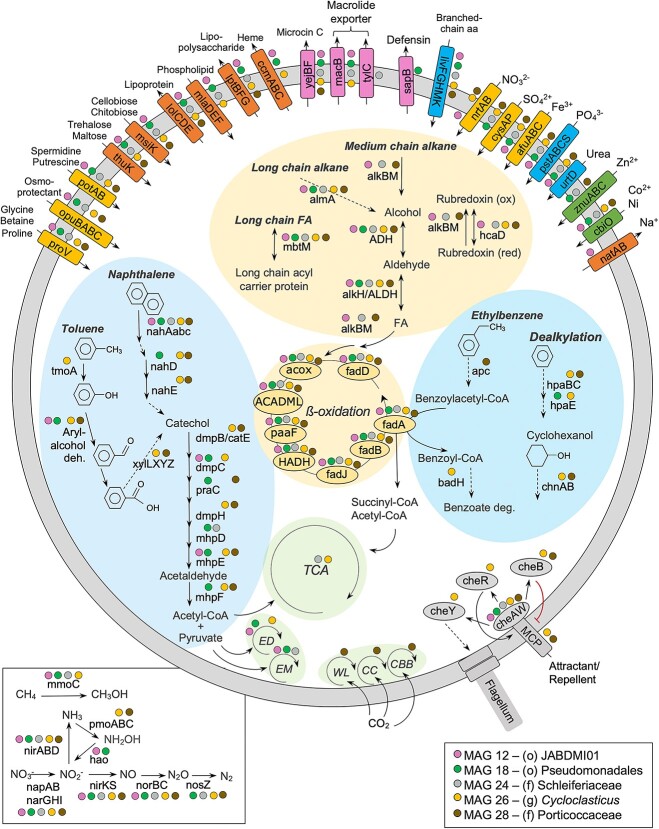
Metabolic pathways of hydrocarbon degradation present in the five selected metagenome-assembled genomes (MAGs). Colored dots indicate predicted pathways and corresponding proteins in a given bacterial genome to demonstrate the comparison between MAGs. The predictions are based on KEGG and CANT-HYD annotations. All five MAGs contained a potential complete set of genes required for alkane and polycyclic aromatic hydrocarbon degradation. Corresponding tables for proteins and pathways are in Tables S9 and S10. Acronyms: Entner–Doudoroff pathway, ED; Embden–Meyerhof pathway, EM; reductive Calvin cycle, CC; reductive Calvin Benson Bassham cycle, CBB; Wood–Ljungdahl pathway, WL; tricarboxylic acid cycle, TCA.

Five MAGs were selected for more in-depth analyses, based on their completeness, contamination, novelty, and on the presence of diverse HB genes, to highlight detailed metabolic pathways of HB degradation (Fig. 8, Table S11). Four of these selected MAGs belong to the *Gammaproteobacteria* (MAG-12, -18, -26, and -28), and MAG-24 belongs to the *Bacteroidota*. Only MAG-26 has a known genus, *Cycloclasticus*, while the four others were classified to the lowest taxonomy level possible (order and family; Table S4). All five selected MAGs possessed a complete set of genes for alkane and/or PAH degradation. The five MAGs can metabolize long-chain alkane and fatty acids, while only three MAGs (MAG-12, -24, and -28) have a complete set of genes for medium chain alkane degradation (Fig. 8). MAG-26 and -28 possessed the most HB genes for several cyclic hydrocarbon degradation pathways. Additionally, all five MAGs possessed three genes for naphthalene 1,2-dioxygenase: the subunit alpha (*nahAa*), the ferredoxin component (*nahAb*, K14578), and the ferredoxin reductase component (*nahAc*, K14579). Several ABC transporters were present in the five MAGs, and only two (MAG-26- and -28) possessed the methyl-accepting chemotaxis protein (MCP) and most other chemotactic genes (*cheABRWY*; K03407, K0341, K00575, K03408, and K03413; Fig. 8, Table S11).

## Discussion

### Community complexity and co-occurrence of multiple-domain microbiomes

The 16S/18S rRNA gene sequencing results indicate that the prokaryotic community composition of the beach sediment differs from that of the coastal seawater (Figs 3 and S1). The beach sediment communities were mainly composed of four genera, including *Granulosicoccus*, *Psychromonas*, *Gillisia*, and *Illuminobacter*. *Granulosicoccus* is an obligate aerobic heterotroph previously found in Antarctic surface seawater [43] and in Antarctic intertidal sediments [44]. *Psychromonas* is a genus commonly found in different polar marine sediments, such as subarctic marine sediment [45], and plays an important role in organic carbon metabolism [46, 47]. *Gillisia* sp*.* constitutes a large fraction of marine bacterioplankton [48] and plays a role in the remineralization of organic matter in the global ocean [49]. *Ilumatobacter* sp. is not endemic to Arctic waters and has been identified in various locations, including coastal sand [50], estuary sediments [51], coastal sediments of the Mediterranean Sea [52], and Arctic deep-sediments [53]. Our study suggests that cold-adapted marine and sediment bacteria were commonly present during the summer months, but most of the bacteria found in our beach sediment and seawater metagenomes were not the classical marine hydrocarbon-degraders (i.e. *Alcanivorax*, *Colwellia*, and *Pseudoalteromonas*) found at other contaminated sites resulting from oil spills (i.e. Exxon Valdez and Deepwater Horizon) [54]. Overall, NWP beach genera exhibited the presence of HB genes (Fig. 6, Tables S7 and S8), which may suggest the potential for these organisms to grow in the event of an oil spill in the NWP environment. Additionally, their HB activity could be enhanced through biostimulation treatments involving nutrient amendments, such as N and P, to the contaminated areas [55].

Interactions between the microbiomes of beach sediments and shoreline seawater are poorly understood. Co-occurrence analyses were used to evaluate and identify patterns among microbial species that may be more difficult to detect using the standard diversity metrics widely used in microbial ecology [56]. Marine cyanobacteria and beach sediment *Bacteroidota* were central to both co-occurrence networks (Figs 4 and 5) and interacted with many members of sediment and marine bacterial hydrocarbon-degraders, including the genera *Granulosicoccus*, *Ilumatobacter*, *Polaribacter*, and *Maribacter*. This suggests that phytoplankton-bacteria and bacteria-bacteria interactions play fundamental roles in marine coastal ecosystems, specifically their contributions to primary production and carbon-nitrogen cycling by uncovering potential relationships of the connections between keystone species [57]. Certain members of the *Flavobacteriales* are recognized as abundant bacterioplankton in non-freshwater environments, and thrive in diverse marine systems, including polar coastal sediments [58] and coastal northern waters [59], as well as open oceans [60]. *Polaribacter* sp. was highly abundant in our samples and represents a prominent fraction in polar oceans [61]. This genus is known to have a tight correlation with phytoplankton blooms in polar regions [60, 62]. This interaction was observed in our co-occurrence network analyses (Fig. 4), where the genus *Polaribacter* connected with several Arctic endemic microbial phytoplankton lineages, including the genera *Mantoniella* (*Chlorophyta*), *Thalassiosira*, *Chaetoceros*, and *Porosira* (*Bacillariophyta*), which dominated the seawater microbial eukaryotic community (Figs 3, S1, and S3). These pico- and nanophytoplankton, together with *Micromonas* sp. (*Chlorophyta*), are commonly found in Arctic waters and constitute a major fraction of annual net primary production [25, 63–65]. These findings highlight the importance of marine phytoplankton in the global community’s interaction with the beach sediment and marine bacterial community. These aforementioned microbial phytoplankton and marine *Flavobacteriales* groups could be selected as keystone or sentinels species, owing to their potential key roles and ecological functions in the Arctic marine ecosystem [66, 67]. This also implies that these groups could be the most competitive groups within this harsh environment, making them important “summer” sentinel species for continuous monitoring of Arctic shorelines in the NWP studies and reflecting environmental indicators of Arctic contaminant exposure across time and space.

### Presence of HB genes in the NWP

Our metagenomes contained a significant number of HB genes, indicating the potential for the coastal community to degrade various alkane and aromatic compounds (Fig. 6). HB genes, including *ahyA*, *alkB*, *almA*, *nahaA*, and CYP153 for alkane degradation, and *fadA*, *ndoB*, non*-ndoB* type, and MAH for aromatic compound degradation, were previously identified in the Arctic Ocean [68, 69], Arctic soils [17, 70], and Arctic beach sediments [24, 71]. Our reconstruction of the MAGs revealed numerous novel microorganism genomes that possess complete alkane degradation pathways and/or cyclic hydrocarbon degradation pathways (Figs 7 and S7), suggesting that the microbial population in the NWP shoreline may utilize hydrocarbon as an alternative carbon source. Moreover, two MAGs, a *Cycloclasticus* sp. and an unclassified *Porticoccaceae*, possess the methyl-accepting chemotaxis protein (MCP; K03406; Fig. 8), which has been shown to play an essential role in the sensing of various substrates, including alkanes [72], and could potentially facilitate the degradation of other hydrocarbons. *Gammaproteobacteria* members, particularly *G. antarcticus*, and *Cycloclasticus*, dominated the high Arctic potential hydrocarbon-degraders community selected by the presence of HB genes, as well as an *Alphaproteobacteria*, *Roseovarius aestuarii*. These lineages are commonly found in seawater and marine sediment [43, 44, 73], and were detected using 16S rRNA gene sequencing and metagenomics from Arctic beach sediments (Resolute and Kivalliq, Nunavut, Canada) [19, 71], indicating ribosomal activity (active cells) at these locations.

### Potential sources of natural hydrocarbons in the NWP

Our study revealed that the coastal core microbiome composition (Figs 3 and S1–S5) and functional annotation of HB genes (Fig. 6) were more diverse and distinct for Dump and Tank Farm beaches compared to the other sites. Both beaches are located near the RB community (Fig. 1), where human activities, such as waste and fuel transportation and storage, and boat refueling, can cause potential hydrocarbon contamination in the RB Harbor. Thus, anthropogenic impacts could alter microbial diversity, causing the pristine microbial diversity of the surrounding area to differ more from the endemic population of the polar environment. This variation may also be influenced by other factors, including the accumulation of hydrocarbons on the sea surface microlayer [74], which can function as a reservoir for these substances, even in remote polar areas, such as the Antarctic [75] and Arctic [76], even where there is no apparent direct origin of hydrocarbon pollution. Other sources of hydrocarbons include direct or indirect inputs, such as biogenic production from microorganisms [77, 78], atmospheric deposition [79], coastal sea-air exchange [80], and Arctic cold subsurface natural seeps, which can occur in the NWP [76, 81].

Marine cyanobacteria are known to widely synthesize hydrocarbons [77], where cyanobacterial alkanes and alkenes are believed to play a significant role in the marine alkane cycle of the upper ocean [18, 82, 83]. Obligate hydrocarbon-degrading bacteria are found in waters without significant levels of petroleum pollution, indicating that these organisms must use an alternate hydrocarbon source [82]. Our data supports this, as *Cyanobacteria* were found to be dominant in our 16S rRNA gene amplicons in all beaches (Figs 3 and S1). In addition, *Cyanobacteria* were co-occurring and correlated with many known hydrocarbon-degrading bacteria [84] and eukaryotic phytoplankton (Fig. 4). However, it is important to note that *Cyanobacteria* are not the only organisms capable of producing hydrocarbons: several eukaryotic phytoplankton, including *Chaetoceros* sp. and *Thalassiosira* sp. [85], and even dinoflagellates, like *Amphidinium* sp. [86], also produce hydrocarbons. These phytoplankton lineages were dominant in our amplicon and metagenomics data (Figs 3, S1, and S3). Marine algae can also produce hydrocarbons like isoprene [87, 88], and may sustain hydrocarbon-degrading bacterial populations in oil-free environments, such as the NWP. The sustainability of biodegradation may be attributed to the advantageous collaboration between coastal microalgae and bacteria, which has synergistic effects on enzymatic reactions and photosynthetic performance [89, 90]. Microalgae also release oxygen through photosynthesis, which can be used by bacteria to oxidize contaminants in the microalgae-bacteria consortium [91]. In return, microalgae benefit from trace elements and nutrients released by bacteria [92].

### Implication of microalgae and viruses in hydrocarbon natural attenuation

To our knowledge, our study is the first to detect HB genes in specific polar marine microalgae and algal viruses. This suggests the potential involvement of coastal phytoplankton as hydrocarbon-degraders. The chlorophyte *Ostreococcus tauri* encoding for the HB gene *nahAa* was detected in our metagenomes (Fig. 6), indicating a potential contribution to HB by marine phytoplankton. Microalgae, specifically green algae from the genera *Selenastrum*, *Scenedemus*, and *Chlorella*, are known to degrade PAHs [93] and play an important role as primary producers in marine ecosystems. They are thought to be essential for PAH degradation in those environments. Such microalgae can reduce PAH bioavailability and toxicity, relying on the production of exopolysaccharides [94, 95], which mediate the uptake of contaminants on the cell surface and/or their complexation into less bioavailable forms [95]. Further in-depth analyses of microbial eukaryotic metagenomic reads and functional annotation may reveal genes involved in the production of exopolysaccharides and their potential involvement in hydrocarbon degradation.

Algal viruses were found in a small proportion in our metagenomes (Fig. S5), and some surprisingly possessed HB genes. We identified the genus *Prymnesiovirus* encoding for genes *almA* groups I and III and *ndoC* (Fig. 6). This algal virus belongs to the *Phycodnaviridae* family, which consists of large double-stranded DNA viruses with a large genome size of up to 560 kb [96]. The *Phycodnaviridae* are considered ecologically important as they infect marine eukaryotic algae [96], as well as harmful phytoplankton species, such as *Phaeocystis* spp. [97] and *Emiliania huxleyi* [98], thus constraining photoautotrophic blooms. It should be noted that significant regulatory communication occurs between phages and mobile genetic elements [99], which is important in the dissemination of valuable genetic material, including HB genes, and in the generation of new catabolic pathways through horizontal gene transfer (HGT) [100, 101]. The presence of HB genes in viruses in our metagenomes, previously undiscovered by studies of Arctic areas, suggests that potential HGT of hydrocarbon genes may play a pivotal role in the evolution of Arctic hydrocarbon-degrading bacterial and phytoplankton populations. It may also contribute to the adaptation of microbial communities to environmental contaminants, by enabling lineages previously incapable of natural attenuation to acquire hydrocarbon degradation capabilities.

### Potential methane aerobic bacterial utilization in NWP beach sediment

Our metagenomes revealed the presence of a small fraction of archaea among the NWP beaches (Fig. S4), including anaerobic methanotrophic archaea (ANME), which are taxonomically related to methanogens and are often detected close to hydrocarbon seeps. The cold subsurface natural hydrocarbon seep at Scott Inlet, Nunavut, in the Canadian Arctic, has reported methane seepage and a high abundance of ANME [76]. Additionally, we observed seven sediment MAGs possessing an incomplete methane oxidation pathway, including unclassified Porticoccaceae (MAG-27, -28, and 29; Fig. S7, Table S9). Upon further analysis of the five selected MAGs, four of them possessed *mmoC* (K16161; Fig. 8, Table S11), a component of methane monooxygenase, while only *Cycloclasticus* (MAG-26) and unclassified Porticoccaceae (MAG-28) possessed *pmoABC* (K10944, K10945, and K10946), a methane/ammonia monooxygenase (Table S11). MAG-28 possessed genes for both aerobic and anaerobic degradation of alkane and aromatic compounds, indicating its potential as a facultative anaerobic bacterium. This suggests the possibility of aerobic methane utilization in the NWP beach sediments, a phenomenon previously observed in specific areas where cold methane-rich fluids leaked from subsurface reservoirs and reached the seafloor and water column in marine environments [102–104], where methane can be oxidized by aerobic methane-oxidizing bacteria. Furthermore, all five selected MAGs contained denitrification genes (Fig. 8), indicating the presence of facultative anaerobic bacterial respiration in the NWP beach sediments. In summary, our study shows that the microbial community in the NWP sediment and seawater has the genetic potential for aerobic methane utilization and hydrocarbon natural attenuation.

## Conclusions

The results provide a comprehensive and detailed screening for the presence of hydrocarbons in the natural attenuation of the Canadian high Arctic NWP coast. Our findings indicate that widely distributed lineages of the *Cyanobacteria*, *Flavobacteriales*, and *Rhodobacterales* may contribute to HB. We observed that some polar microbial phytoplankton hydrocarbon-degraders and the dissemination of HB genes by HGT from algal viruses may play a role in this process. Furthermore, our results suggest the presence of methane-oxidation in aerobic bacteria in NWP beach sediments. The application of metagenomics and environmental DNA metabarcoding has facilitated our understanding of the biodiversity, functionality, and ecology of the beach and shoreline communities in the context of oil natural attenuation. Although, these multi-omics results must be corroborated by culture-dependent methods, metatranscriptomics, and DNA stable isotope probing enrichment to confirm the capacity of marine polar and beach sediment strains to metabolize hydrocarbons or produce them under aerobic and anaerobic conditions. The integration of this knowledge will facilitate future monitoring of the effectiveness of bioremediation and/or natural attenuation processes for contaminated shorelines. It will facilitate the development of a modeling approach to help simulate and predict the responses of such biodegradative microorganisms during bioremediation treatments in the event of future hydrocarbon spills in the NWP.

## Supplementary Material

ISMECOMMUN-D-24-00102_suppl_methods_tables_figures_20240711_ycae100

ISMECOMMUN-D-24-00102_Supplementary_tables_20240612_ycae100

## Data Availability

All amplicon raw sequences and long-read sequences from Oxford nanopore MinION are in the NCBI GenBank Sequence read Archive (SRA) under BioProject PRJNA1073184, SRA36334601-SRA36334631, and SRA36335098-SRA36335121, respectively.

## Code availability

This paper does not report original code. All code was adapted from the user manual of the software packages.
