# Supplementary material for: High Arctic seawater and coastal soil microbiome co-occurrence and composition structure and their potential hydrocarbon biodegradation
Source: ISME Commun. 2024 Jul 16;4(1):ycae100. doi: 10.1093/ismeco/ycae100 (PMC11296632; doi:10.1093/ismeco/ycae100)
Supplement: ISMECOMMUN-D-24-00102_suppl_methods_tables_figures_20240711_ycae100 [file ismecommun-d-24-00102_suppl_methods_tables_figures_20240711_ycae100.docx]

**High Arctic Seawater and Coastal Soil Microbiome Co-occurrence and Composition Structure and Their Potential Hydrocarbon Biodegradation**

**Nastasia J. Freyria^1*^, Esteban Góngora^1^, Charles W. Greer^1,2^ & Lyle G. Whyte^1^**

^1^Department of Natural Resource Sciences, Faculty of Agricultural and Environmental Sciences, McGill University, 21 111 Lakeshore Road, Macdonald Stewart Building, Room MS3-053, Ste. Anne-de-Bellevue, Quebec, H9X 3V9, Canada

^2^Energy, Mining and Environment Research Centre, National Research Council Canada, 6100 Royalmount Ave., Montreal, QC, H4P 2R2, Canada

*** Correspondence:**Corresponding Author
[nastasia.freyria@mcgill.ca](mailto:nastasia.freyria@mcgill.ca)

**SUPPLEMENTARY METHODS**

**Site description and sample collection**

Resolute Bay is situated in the Canadian high Arctic Archipelago, specifically on Cornwallis Island in Nunavut, Canada. Beach sediment samples were collected from five intertidal zones during the summers of 2018, 2019, 2021, and 2022 (**Table S1**). The five selected beaches, namely Assistance Bay, Dump Beach, Dynamite Beach, Tank Farm and Tupirvik Beach, are situated along Resolute shoreline facing the Northwest Passage (**Fig. 1**). Three of the beaches were considered pristine (Tupirvik, Dynamite and Assistance Bay), while two were in areas that had human activity (Dump and Tank Farm). Tank Farm Beach samples were collected from an area adjacent to the Resolute Bay village tank farm which stores heating fuel and is replenished annually, while the Resolute Bay dump beach is ~75 m away from the municipal waste dump. Assistance Bay is located southeast of the Inuit community near Tank Farm beach. Resolute is in a region that remains covered in ice for at least 10 months out of the year. We added seawater sampling of Assistance Bay lagoon, which a small, enclosed crick that is located at Assistance Bay, but not facing the Northwest Passage. According to historical data from Climate Weather Canada (<https://climate.weather.gc.ca/historical_data>), the average daily temperature in July and August is 4.5°C and 2°C, respectively.

**Sample collection**

To gain a comprehensive understanding of the composition and abundance of the microbial community, we conducted a five-year study, sampling beach sediment over the course of four consecutive summers (2018 to 2022), with the exception of 2020 due to the COVID pandemic when our planned field trip was cancelled. A total of 41 samples were gathered from the intertidal zone of Resolute coastline, comprising 19 sediment samples over the 4 summers of 2018, 2019, 2021 and 2022, and 22 surface seawater samples of only summer 2022. Aseptic sampling of beach sediments was conducted by digging approximately 5 cm into the ground using sterilized collection tools treated with 70% ethanol prior to use. The samples were loaded into sterile Falcon tubes (Corning Inc., Corning, NY, USA) and Nasco Whirl-Pak collection bags (Sigma-Aldrich, Oakville, ON, Canada). Approximately 4 L of coastal surface seawater were collected from each coastal site. The samples were transported to the laboratory of the Polar Continental Shelf Program facility (PCSP), situated close to the Inuit community (**Fig. 1**). Sediment samples were immediately stored at PCSP at -80°C.

Seawater was filtered for size collection using a peristaltic pump (MityFlex, Anko, Bradenton, FL, USA) equipped with a 0.2 *µ*m polycarbonate filter (PC, AMD Manufacturing Inc., Mississauga, ON, Canada) for no more than 3 h to avoid RNA loss from cells. Two PC filters were used to filtrate approximately 4 L of seawater. After filtration, the PC filters were placed in a 2 mL cryovial tube. Additionally, 250 mL of surface seawater was filtered twice in-situ at each site through a 0.2 *µ*m Sterivex Unit (Millipore, Sigma-Aldrich) using a sterile 50 mL syringe (Luer-Lock Syringe, Thermo Fischer Scientific, Waltham, MA, USA) to compare both filtration methods. For each method, 1.8 mL of RNAlater (Ambio, ThermoFisher Scientific) was added to the Sterivex and cryovial tubes to preserve the RNA. After being kept in a buffer at room temperature for 1 h, all tubes were stored at -80°C for further analysis. Subsequently, the samples were transported in coolers to McGill University in Montréal, Québec, Canada and stored at -80°C until analyzed.

**Environmental variables measurements**

A volume of 25 mL of coastal surface seawater was sampled at each site for measuring nitrate and phosphate concentrations using CHEMetrics Inc. test kits (K-8503 and k-6903) with the V-2000 CHEMtrics photometer. In-situ measurements of shoreline seawater were taken using YSI ProQuatro Multiparameter Instrument (Xylem Inc.) to determine seawater temperature, salinity, and pH. Dissolved oxygen was also measured in situ with a PyroScience Picco-2 oxygen meter (Aachen, Germany).

**Seawater microbial cell concentrations and flow cytometry**

To assess the abundance of marine microbial phytoplankton and marine bacteria, we collected coastal surface seawater in a 10 mL sterile Falcon tube. Then, we added 90 *µ*L of 25% glutaraldehyde to 1.8 mL of seawater in a 2 mL cryovial tube, resulting in a final concentration of 1% (v/v). After 30 min of cell fixation at 4°C in the dark, we stored the samples at -80°C until laboratory analysis. To quantify bacterial counts, 200 *µ*L of fixed samples were stained by adding 0.5 *µ*L of Sybr Green I (Invitrogen, Thermo Fisher Scientific) from a 1000 x stock solution. To enumerate bacterial cells, we utilized a BD Accuri C6 flow cytometer (BD Biosciences, Franklin Lakes, NJ, USA) equipped with a CSampler at Institut de Biologie Intégrative et des Systèmes, Laval University, Québec, Canada (**Table S2**). The methods outlined in Freyria et al. (2022) were followed for enumeration, while the data acquisition method was based on (Belzile et al., 2008; Marie et al., 2000). Briefly, to quantify bacterial cells, 200 *µ*L of each fixed and stained sample were run at a slow flow rate of 73 *µ*L.min^-1^ for 5 min. To enumerate phytoplankton cells, 800 *µ*L of each fixed sample was run at a slow flow rate of 81 *µ*L.min^-1^ for 10 min. Each run underwent three agitation and three wash cycles between each sample and was recalibrated with 2 *µ*m Fluoresbrite beads (BD Trucount, Biosciences) in filtered seawater to normalize cell counts with flow rate. As previously described by Freyria et al. (2022), eukaryotic pico- and nanophytoplankton were differentiated using chlorophyll red fluorescence and side scattered light at 670 nm.

**DNA and RNA extraction**

DNA was extracted from 0.5 g of beach sediment using the DNeasy PowerLyzer PowerSoil kit (Qiagen, Hilden, Germany) according to the manufacturer’s protocol, eluted in 30 *µ*L of nuclease-free water, and then stored at -20°C until library preparation. DNA and RNA were extracted from surface seawater samples of PC filters and Sterivex using the All-Prep DNA/RNA Mini Kit (Qiagen) and eluted in 30 *µ*L of nuclease-free water as described previously by Freyria et al. (2021). The HighCapacity Reverse Transcription Kit (Applied Biosystems, Thermo Fischer Scientific) was used to convert RNA to cDNA, following the manufacturer’s suggestions.

**Library preparation and sequencing**

Prokaryotic primers (515F/926R) coupled with Illumina adapters were used to target the V4 region of 16S rRNA gene in DNA sediment and in DNA and cDNA seawater samples, following the methodology described in Parada et al. (2016). Similarly, the V4 region of 18S rRNA in both DNA and cDNA seawater samples was targeted using eukaryotic specific primers (E572F/E1009R) coupled with Illumina adaptors, and all prepared samples underwent nested PCR using the conditions described by Comeau et al. (2011) for 18S rRNA gene sequencing and as in Magnuson et al. (2023) for 16S rRNA gene sequencing. The resulting PCR products were purified using Sera-Mag Select bead clean-up (Cytiva, Burnaby, BC, Canada) and quantified using a NanoDrop One C spectrophotometer (Thermo Fischer Scientific), and a Qubit v4 fluorometer (Life technologies, Thermo Fischer Scientific) with the 1X dsDNA High Assay Kit (Invitrogen). The DNA libraries were then pooled equimolarly for normalization. The quality and quantity of the pooled library were verified using the Agilent High Sensitivity DNA kit on a 2100 Bioanalyzer (Agilent Technologies, Santa Clara, CA, USA). The indexed and pooled final library was sequenced in-house using the 600-cycle MiSeq Reagent Kit v3 on an Illumina MiSeq platform. DNA extracts from sediment samples and seawater were prepared for sequencing using the Rapid PCR Barcoding kit (SQK-RPB004m Oxford Nanopore Technologies) following the manufacturer’s protocol. Purification was conducted with AMPure XP beads (Beckman Coulter Life Sciences, Brea, CA, USA). The indexed library was quantified using a Qubit v4 fluorometer and the Qubit 1X dsDNA High Sensitivity Assay Kit (Invitrogen). In-house sequencing was performed using a MinION Mk1c (MC-110217, Oxford Nanopore Technologies, Oxford, England) device on separate R9.4 FLO-MIN106 flow cells (Oxford Nanopore Technologies).

**Sequence data processing**

To generate amplicon sequence variants (ASVs), all 16S and 18S rRNA gene sequences were processed using R v4.1.2 and the R package *dada2* v1.22.0 (Callahan et al., 2016). Taxonomy was assigned to the ASVs using the Silva Reference Database v138 (Callahan, 2018) and the Protist Ribosomal Reference (PR^2^) database v4.110 (Guillou et al., 2012). AVSs matrix was filtered and rarefied according to Freyria et al. (2021). AVSs classified as metazoan and fungi were eliminated, and rarefaction was performed with QIIME 2 (Bolyen et al., 2018) based on the sample with the lowest number of reads. Among the eukaryotic community, we retained a total of 1 213 641 reads, including 731 641 of 18S rRNA gene (DNA) reads and 481 628 of 18S rRNA (transcriptional products – RNA) reads. These reads were then clustered into a total of 1 839 ASVs (at 98% similarity), with 1 291 ASVs (DNA) and 1 246 ASVs (RNA). The 16S AVS matrix was also rarefied using the same approach as described above. We obtained a total of 19 449 ASVs with a total of 3 231 367 reads, with 2 475 972 reads (DNA dataset) and 755 395 reads (RNA dataset). The ASVs tables of 18S and 16S rRNA gene amplicon seawater samples, the average of each duplicate was calculated to analyze the taxonomy and relative abundance.

All MinION sequences were basecalled and adapter trimmed using MinKNOW v20.03.5 and MinKNOW GUI v0.1.8. The basecalled sequences were demultiplexed and assembled using Flye v2.9-b1774 (Kolmogorov et al., 2020) with default parameters and “nano-hq”. Following assembly, MetaErg v1.2.0 (Dong et al., 2018) was used to annotate the contigs. This included identifying and annotating genes using the KEGG and PFAM databases, as well as taxonomic assignment of genes using SILVA database v132 (Quast et al., 2012). Carbohydrate-active enzymes (CAZyme) were predicted using dbCAN (Huang et al., 2018), a meta server for automated CAZyme annotation through Hidden Markov Model HMMER search with hmmscan v3.3.2 (Finn et al., 2011) through the CAZy database (Cantarel et al., 2009). The Calgary approach to annotating hydrocarbon (CANT-HYD) (Khot et al., 2022) was utilized to detect the presence of 37 crucial marker genes among all Nanopore reads, with a ‘noise’ cut-off score. The CANT-HYD enables the identification of genes involved in both aerobic and anaerobic hydrocarbon degradation pathways, encompassing aliphatic and aromatic hydrocarbons. To eliminate the technical bias associated with different depths of metagenomic sequencing effort, we calculated the count of gene x per the total number of reads and we multiplied it by a million to facilitate interpretation.

We performed metagenomic binning of each of the 24 metagenome assemblies individually to reconstruct metagenome-assembled genomes (MAGs; **Table S3**). Multiple binning tools, including MetBAT2 v2.15 (Kang et al., 2019), MaxBin2 v2.2.7 (Wu et al., 2014), and concoct v1.1.0 (Alneberg et al., 2013), were used for genome binning to increase the number of bins. Only contigs longer than 1500 bp were retained. The completeness and contamination level of all bins were assessed using CheckM v1.1.3 (Parks et al., 2015), and only bins with a contamination level below 15% and a completeness greater than 50% were kept, resulting in a total of 63 filtered medium quality MAGs (**Table S4**). The replication of bins was verified using dRep v3.2.2 (Olm et al., 2017), resulting in a total of 63 medium-quality bins. Taxonomy determination for the bins was done using MetaErg and the Genome Taxonomy Database Toolkit (GTDB-Tk) v2.1.0 (Chaumeil et al., 2020). The analyses were conducted using Compute Canada facilities and in-house computers. The gene count in each metagenome was normalized by hits per million genes based on the count of genes divided by the total number of reads multiplied by a million.

**Statistical analyses**

The R packages *ggplot2* v3.4.3 (Wickham, 2011), and *dplyr* v1.0.9 (Wickham et al., 2019) were used to generate plots. The Resolute map was created using Ocean Data View v5.6.2 (Schlitzer, 2015). Packed circle plots were created using the R package *packcircles* v0.3.5 (Bedward and Eppstein, 2018) (**Fig. 3**). Beta diversity was analyzed using Constrained Correspondence Analysis (CCA) ordination with the R package *vegan* v2.6.2 (Oksanen, 2013). CCA ordination allows the differentiation between sampling stations based on the most significant explained variance in environmental factors (**Fig. 2**). To assess the significance of CCA constraints, we performed an ANOVA-like permutation test for constrained correspondence analysis using the function *anova()* from the R package *vegan.* A permutational multivariate analysis of variance (PERMANOVA) using distance matrices was employed using the function *adonis2()* from the R package *vegan* to ascertain noteworthy effects of environmental factors with the combined community (**Table S5**). The function *p.adjust()* from the R package *stats* v. 4.3.3 was applied to generate adjusted *p-*value with the Benjamin-Hochberg method. Spearman’s rank correlation analysis was conducted using the R packages *corrplot* v0.92 (Wei et al., 2017) and *corrgram* v1.14 (Wright and Wright, 2018) to examine potential associations between environmental factors and cell concentration of marine prokaryotes and microbial eukaryotes, based on flow cytometry data (**Fig. 2, Table S2**).

Additionally, the top 50 most abundant ASVs from the three sample categories, including 18S of seawater, 16S of seawater and 16S of sediment, were selected to identify specific trends determined by the z-score calculation based on the method previously described by Eiler et al. (2012). Briefly, the z-score is calculated by subtracting the relative abundance of an ASV from the ASV’s mean relative abundance in a sample, then dividing it by the standard deviation (**Fig. S1**). The z-score is advantageous for comparing the structure and dynamic community because it incorporates both the mean and standard deviation for standardization (Legendre and Legendre, 1998).

The CoNet plugin (Faust and Raes, 2016) in Cytoscape v3.9.1 (Shannon et al., 2003) was used to construct co-occurrence networks solely from three separate ASVs tables of only the top 500 most abundant ASVs of sediment prokaryotes, marine prokaryotes, and marine microbial eukaryotes in the relative abundance table of rDNA (**Figs. 4-5**). The purpose was to compare associations among marine eukaryotes with those among marine prokaryotes, and among marine prokaryotes with sediment prokaryotes. The two correlation networks were computed using five distinct methods: three rank correlations from Kendall, Pearson, and Spearman; one method that calculates similarity based on the distance between probability distributions (mutual information); and one method based on Bray-Curtis dissimilarity distance. Randomized scores were computed using 1000 permutations and bootstrap with 1000 iterations. Brown’s method (Brown and Russell, 1997) was employed to measure specific *p*-value scores. False-positive associations were eliminated from the final network using the Benjamini-Hochberg correction method with a *p*-value threshold of less than 0.05 (Thissen et al., 2002).

| **Sampling date** | **Latitude (°N)** | **Longitude (°W)** | **Temp.** | **Sal.** | **DO** | **pH** | **Nitrate** | **Phosphate** | **Avg air temp. (°C)** | **Sea ice conc. (%)** |
| --- | --- | --- | --- | --- | --- | --- | --- | --- | --- | --- |
| ***Assistance Bay Beach*** | |  |  |  |  |  |  |  |  |  |
| 09 July 2019 | 74.6507 | -94.2977 | 4.2 | 0.01 | 14.54 | n.d. | n.d. | n.d. | 6.5 | 90 |
| 07 Aug.2021 | 74.6483 | -94.2930 | 4.8 | 3.18 | 10.56 | 8.15 | 0.02 | 0.02 | 10.5 | 40 |
| 28 July 2022 | 74.6480 | -94.2927 | 2.7 | 2.67 | 5.40 | 9.13 | 0.24 | 5.25 | 6.5 | 45 |
|  |  |  |  |  |  |  |  |  |  |  |
| ***Assistance Bay lagoon*** | |  |  |  |  |  |  |  |  |  |
| 28 July 2022 | 74.6482 | -94.2919 | 1.7 | 1.80 | 10 | 10.59 | 0.08 | 0 | 6.5 | 0 |
|  |  |  |  |  |  |  |  |  |  |  |
| ***Dump Beach*** |  |  |  |  |  |  |  |  |  |  |
| 08 July 2018 | 74.6716 | -94.9229 | n.d. | n.d. | n.d. | n.d. | n.d. | n.d. | 4.5 | 35 |
| 08 July 2019 | 74.6718 | -94.9249 | 0.1 | 0.51 | 15.26 | 6.1 | 0.05 | 0.13 | 4 | 80 |
| 06 Aug. 2021 | 74.6708 | -94.9211 | 2.1 | 3.20 | 10.69 | 7.98 | 0.02 | 0.76 | 9 | 45 |
| 27 July 2022 | 74.6716 | -94.9229 | -0.2 | 2.69 | 10 | 9.2 | 0.07 | 4.77 | 1 | 45 |
|  |  |  |  |  |  |  |  |  |  |  |
| ***Dynamite Beach*** | |  |  |  |  |  |  |  |  |  |
| 08 July 2018 | 74.7023 | -94.0822 | n.d. | n.d. | n.d. | n.d. | n.d. | n.d. | 3 | 35 |
| 08 July 2019 | 74.7064 | -95.0785 | 1.9 | 0.45 | 14.21 | 6.1 | 0.13 | 0.09 | 4 | 80 |
| 06 Aug. 2021 | 74.7019 | -95.0825 | 1.1 | 3.11 | 10.55 | 8 | 0.02 | 0.33 | 9 | 45 |
| 25 July 2022 | 74.7022 | -95.0824 | 1.4 | 2.69 | 5.57 | 9.15 | 0.07 | 0 | 8 | 45 |
|  |  |  |  |  |  |  |  |  |  |  |
| ***Tank* *Farm Beach*** | |  |  |  |  |  |  |  |  |  |
| 08 July 2018 | 74.6845 | -94.8888 | n.d. | n.d. | n.d. | n.d. | n.d. | n.d. | 3 | 35 |
| 08 July 2019 | 74.6845 | -94.8888 | 1.5 | 0.07 | 13.06 | 6.1 | 0.05 | 0 | 4 | 80 |
| 06 Aug. 2021 | 74.6845 | -94.8890 | 2.3 | 3.15 | 9.99 | 8.21 | 0.02 | 1.09 | 9 | 45 |
| 27 July 2022 | 74.6845 | -94.8889 | 3.3 | 0.22 | 9 | 9.2 | 0.07 | 0 | 1 | 45 |
|  |  |  |  |  |  |  |  |  |  |  |
| ***Tupirvik Beach*** | |  |  |  |  |  |  |  |  |  |
| 08 July 2018 | 74.7437 | -95.0487 | n.d. | n.d. | n.d. | n.d. | n.d. | n.d. | 4.5 | 35 |
| 08 July 2019 | 74.7435 | -95.0486 | 4.5 | 0.19 | 13.65 | 6.5 | 0.08 | 0 | 4 | 80 |
| 05 Aug. 2021 | 74.7434 | -95.0485 | 1.3 | 3.24 | 10.25 | 8.2 | 0.005 | 1.18 | 9.5 | 40 |
| 25 July 2022 | 74.7436 | -95.0487 | 1.1 | 1.13 | 7.22 | 9 | 0.08 | 0 | 8 | 45 |
|  |  |  |  |  |  |  |  |  |  |  |

**Table S1.** Stations sampled during summers 2018, 2019, 2021 and 2022. Abbreviations: Temp. – seawater temperature (°C), Sal. – seawater salinity (%), DO – seawater dissolved oxygen (mg.L^-1^). Nitrate and phosphate concentration were measured in coastal surface seawater (ppm). Sea ice concentration was determined from a visual analysis of ice charts produced by the Canadian Ice Service and images from National Snow and Ice Data Center and NASA.

No data – n.d.

**Table S2 (xlsx)**. Flow cytometry cell enumeration (cells.mL^-1^) for seawater samples of 2022.

**Table S3.** Overall results of Oxford Nanopore MinION sequencing.

| **Year** | **Type of sample** | **Raw reads** | **Contigs** |
| --- | --- | --- | --- |
| ***Assistance Bay*** |  |  |  |
| 2019 | Sediment | 629895 | 1902 |
| 2021 | Sediment | 3160764 | 10915 |
| 2022 | Sediment | 946074 | 4010 |
| 2022 | Seawater | 908546 | 4713 |
| ***Dump Beach*** |  |  |  |
| 2018 | Sediment | 591810 | 2702 |
| 2019 | Sediment | 1720551 | 7203 |
| 2021 | Sediment | 1111965 | 37627 |
| 2022 | Sediment | 644214 | 1694 |
| 2022 | Seawater | 505035 | 2086 |
| ***Dynamite Beach*** |  |  |  |
| 2018 | Sediment | 563613 | 4465 |
| 2019 | Sediment | 106325 | 6031 |
| 2021 | Sediment | 1992991 | 9742 |
| 2022 | Sediment | 896810 | 2347 |
| 2022 | Seawater | 4538246 | 14284 |
| ***Tank Farm Beach*** |  |  |  |
| 2018 | Sediment | 804550 | 14411 |
| 2019 | Sediment | 166908 | 274 |
| 2021 | Sediment | 1560228 | 6151 |
| 2022 | Sediment | 842080 | 1506 |
| 2022 | Seawater | 4018786 | 5629 |
| ***Tupirvik Beach*** |  |  |  |
| 2018 | Sediment | 410903 | 6943 |
| 2019 | Sediment | 77044 | 3800 |
| 2021 | Sediment | 186485 | 1435 |
| 2022 | Sediment | 460805 | 3135 |
| 2022 | Seawater | 3398319 | 5635 |

**Table S4 (xlsx).** Metagenome-assembled genomes (MAG) supplemental information.

**Table S5.** Permutational multivariate analyses of variation using distance matrices (PerMANOVA) test comparing environmental factors of sediment surface seawater of all samples taken along Resolute shoreline. Temperature (°C), dissolved oxygen (DO, mg.L^-1^), nitrate concentrations (ppm), sum of squares (SumOfSqs), pseudo-F ratio (F) and Pr(>F), where *p*-value is based on Monte Carlo random draws and was generated based on 999 permutations. All *p-*value were adjusted (adj.p-value) using the Benjamin-Hochberg method.

|  | **Coastal sediment and seawater of 16S community** | | | | | |
| --- | --- | --- | --- | --- | --- | --- |
|  | **Df** | **SumOfSqs** | **R^2^** | **F** | **Pr(>F)** | **Adj.p-value** |
| **Temperature** | 1 | 0.073 | 0.01 | 0.26 | 0.98 | 0.99 |
| **Salinity** | 1 | 0.31 | 0.08 | 1.14 | 0.30 | 0.99 |
| **DO** | 1 | 0.12 | 0.03 | 0.45 | 0.81 | 0.99 |
| **pH** | 1 | 0.15 | 0.04 | 0.55 | 0.72 | 0.99 |
| **Nitrate** | 1 | 0.07 | 0.01 | 0.25 | 0.99 | 0.99 |
| **Residual** | 11 | 3.96 | 0.80 |  |  |  |
| **Total** | 16 | 3.81 | 1.00 |  |  |  |
|  |  |  |  |  |  |  |
|  | **Coastal seawater of 16S community** | | | | | |
| **Temperature** | 1 | 0.04 | 0.03 | 0.38 | 0.94 | 0.98 |
| **Salinity** | 1 | 0,.33 | 0.26 | 2.76 | 0.042 * | 0.21 |
| **DO** | 1 | 0.07 | 0.05 | 0.61 | 0.73 | 0.98 |
| **pH** | 1 | 0.03 | 0.03 | 0.32 | 0.98 | 0.98 |
| **Nitrate** | 1 | 0.05 | 0.04 | 0.42 | 0.91 | 0.98 |
| **Residual** | 6 | 0.73 | 0.57 |  |  |  |
| **Total** | 11 | 1.28 | 1.00 |  |  |  |
|  |  |  |  |  |  |  |
|  | **Coastal seawater of 18S community** | | | | | |
| **Temperature** | 1 | 0.36 | 0.06 | 3.08 | 0.03 * | 0.08 |
| **Salinity** | 1 | 1.36 | 0.22 | 11.43 | 0.001 *** | 0.004 ** |
| **DO** | 1 | 0.71 | 0.11 | 5.99 | 0.001 *** | 0.004 ** |
| **pH** | 1 | 0.15 | 0.25 | 1.26 | 0.25 | 0.25 |
| **Nitrate** | 1 | 0.30 | 0.05 | 2.57 | 0.04 * | 0.08 |
| **Residual** | 26 | 3.10 | 0.51 |  |  |  |
| **Total** | 31 | 6.02 | 1.00 |  |  |  |

*Significant *p*-values. Tests are based on Bray-Curtis dissimilarity distances and 999 permutations.

**Table S6.** Hydrocarbon biodegradative genes of alkane and aromatic compound degradation found in metagenomes and metagenome-assembled genomes from **Figs. 6-7 and S7**. Symbol x in CANT-HYD column represents the presence of the gene in the database.

| **Gene name** | **KEGG: KO** | **CANT-HYD** | **Enzyme** |
| --- | --- | --- | --- |
| ***Aerobic alkane degradation*** | |  |  |
| AlkB | K00496 | x | AlkB-type alkane hydroxylase |
| AlmA GroupI |  | x | Alkane monooxygenase |
| AlmA GroupIII |  | x | Alkane monooxygenase |
| CYP153 |  | x | Cytochrome P450 alkane hydroxylase |
| LadA alpha | K20938 | x | Long-chain alkane monooxygenase |
| LadA beta |  | x | Long-chain alkane monooxygenase |
| LadB | K04091, K20938 | x | Long-chain alkane monooxygenase |
| pBmoA | K10944 | x | butane monooxygenase (particulate) |
| pBmoB | K10945 | x | butane monooxygenase (particulate) |
| pBmoC | K10946 | x | butane monooxygenase (particulate) |
| PrmA | K18223 | x | Propane 2-monoxygenase |
| PrmC | K18224 | x | Propane 2-monoxygenase |
| sBmoX | K16157 | x | butane monooxygenase (soluble) complex |
| sBmoY | K16158 | x | butane monooxygenase (soluble) complex |
| sBmoZ | K16159 | x | butane monooxygenase (soluble) complex |
| ***Aerobic aromatic degradation*** | |  |  |
| CatA | K03381 |  | Catechol 1,2-dioxygenase |
| CatE | K07104 |  | Catechol 2,3-dioxygenase |
| DmpO | K16245 | x | Phenol hydroxylase |
| DszC | K22219 | x | Dibenzothiophene desulfurization enzyme C |
| MAH_alpha | K03268, K05708, K08689, K14748 | x | Monoaromatic dioxygenase alpha subunit |
| MAH_beta | K14580, K15750, K16268 | x | Monoaromatic dioxygenase beta subunit |
| NahAa | K14581 |  | Naphthalene 1,2-dioxygenase ferredoxin reductase component |
| NahAb | K14578 |  | Naphthalene 1,2-dioxygenase ferredoxin component |
| NahAc | K14579 |  | Naphthalene 1,2-dioxygenase subunit alpha |
| NidA | K11943 |  | PAH dioxygenase large subunit |
| NdoB | K14579 | x | Naphthalene 1,2-dioxygenase alpha |
| NdoC | K14579 | x | Naphthalene 1,2-dioxygenase beta subunit |
| non NdoB type |  | x | Naphthalene 1,2-dioxygenase alpha |
| TmoA BmoA | K15760 | x | Toluene 4-monooxygenase |
| TmoB BmoB | K15761 | x | Toluene 4-monooxygenase |
| TmoE | K15764 | x | Toluene 4-monooxygenase |
| TomA1 | K15764, K16243 | x | Toluene 2-monooxygenase/Phenol Hydroxylase |
| TomA3 | K16242 | x | Toluene 2-monooxygenase/Phenol Hydroxylase |
| TomA4 | K16245 | x | Toluene 2-monooxygenase/Phenol Hydroxylase |
| XylA | K15758 |  | Toluene methyl-monooxygenase electron transfer component |
| ***Anaerobic alkane degradation*** | |  |  |
| AhyA |  | x | Alkane C2 methylene hydroxylase (putative) |
| AssA | K00656, K07540 | x | 1-methylalkyl (alkyl) succinate synthase |
| ***Anaerobic aromatic degradation*** | |  |  |
| AbcA_1 | K03182 | x | Benzene carboxylase |
| AbcA_2 |  | x | Benzene carboxylase |
| Apc | K10701 |  | Acetophenone carboxylase |
| BadH | K07535 |  | 2-hydroxycyclohexanecarboxyl-CoA dehydrogenase |
| BsdC | K01612 |  | vanillate/4-hydroxybenzoate decarboxylase subunit C |
| BsdD | K21759 |  | vanillate/4-hydroxybenzoate decarboxylase subunit D |
| BssA | K00656, K07540 | x | Benzyl/(4-Isopropylbenzyl) succinate synthase |
| BbsG | K07545 |  | Benenzyzlysluscuccicniynly-CoA dehydrogenase |
| CmdA | K10700, K17050 | x | p-cymene dehydrogenase |
| EbdA | K10700 | x | Ethylbenzene dehydrogenase |
| FadA | K00632 |  | Acetyl-CoA acyltransferase |
| HbaA | K04105 |  | 4-hydroxybenzoate-CoA ligase |
| K27540 |  | x | Naphthalene carboxylase |
| NmsA | K07540 | x | Naphthyl 2-methylsuccinate synthase |
| Ped | K14746 |  | (S)-1-phenylethanol dehydrogenase |

**Table S7 (xlsx).** List of all hydrocarbon biodegradative genes present in each beach and taxonomy at the phylum level that possess hydrocarbon degradation genes from **Fig. 6**. Counts per sample were normalized to log hits per million (hpm) coding genes.

**Table S8.** Top 10 hydrocarbon-degraders taxa at the lowest level of taxonomy across the five Canadian High Arctic beaches from **Fig. S6**. Relative abundance is based on the number of reads for each gene with a known hydrocarbon degradation annotation for each site.

| **Phylum** | **Class** | **Order** | **Lowest level of taxonomy** | **Relative abundance (%)** |
| --- | --- | --- | --- | --- |
| ***Assistance Bay Beach*** | |  |  |  |
| Proteobacteria | Gammaproteobacteria | Granulosicoccales | (s) *Granulosicoccus antarcticus* | 28.69 |
| Proteobacteria | Gammaproteobacteria | Methylococcales | (g) *Cycloclasticus* sp. | 3.85 |
| Proteobacteria | Alphaproteobacteria | Rhodobacterales | (s) *Roseovarius aestuarii* | 2.78 |
| Proteobacteria | Gammaproteobacteria | Pseudomonadales | (g) ASP10-02a | 2.57 |
| Proteobacteria | Alphaproteobacteria | Rhodobacterales | (g) UBA3435 | 2.36 |
| Proteobacteria | Gammaproteobacteria | Pseudomonadales | (g) HTCC2207 | 1.82 |
| Proteobacteria | Gammaproteobacteria | Pseudomonadales | (g) HTCC2207 | 1.71 |
| Bacteroidota | Bacteroidia | Flavobacteriales | (g) SCGC-AAA160-P02 | 1.39 |
| Proteobacteria | Gammaproteobacteria | Pseudomonadales | (g) HTCC2207 | 1.39 |
| Proteobacteria | Alphaproteobacteria | Rhodobacterales | (g) HLUCCA08 | 1.18 |
| Proteobacteria | Alphaproteobacteria | Rhodobacterales | (g) *Planktomarina* sp. | 1.18 |
|  |  |  |  |  |
| ***Dump Beach*** |  |  |  |  |
| Proteobacteria | Gammaproteobacteria | Granulosicoccales | (s) *Granulosicoccus antarcticus* | 21.43 |
| Proteobacteria | Alphaproteobacteria | Rhodobacterales | (s) *Roseovarius aestuarii* | 2.15 |
| Proteobacteria | Gammaproteobacteria | Granulosicoccales | (g) GCA-1730015 | 1.15 |
| Proteobacteria | Gammaproteobacteria | Enterobacterales | (s) *Zobellella* A *maritima* | 1.04 |
| Proteobacteria | Alphaproteobacteria | Rhizobiales | (g) *Labrenzia* sp. | 0.67 |
| Proteobacteria | Alphaproteobacteria | Rhodobacterales | (g) HLUCCA08 | 0.67 |
| Proteobacteria | Alphaproteobacteria | Rhodobacterales | (s) *Thalassococcus halodurans* | 0.67 |
| Bacteroidota | Bacteroidia | Flavobacteriales | (s) *Pricia antarctica* | 0.59 |
| Proteobacteria | Gammaproteobacteria | Pseudomonadales | (g) *Halioglobus* sp. | 0.59 |
| Proteobacteria | Gammaproteobacteria | UBA10353 | (f) UBA7415 | 0.59 |
|  |  |  |  |  |
| ***Dynamite Beach*** |  |  |  |  |
| Proteobacteria | Gammaproteobacteria | Granulosicoccales | (s) *Granulosicoccus antarcticus* | 23.48 |
| Proteobacteria | Gammaproteobacteria | Pseudomonadales | (g) HTCC2207 | 2.00 |
| Proteobacteria | Gammaproteobacteria | Pseudomonadales | (g) ASP10-02a | 1.81 |
| Actinobacteriota | Acidimicrobiia | Microtrichales | (s) *Ilumatobacter nonamiensis* | 1.48 |
| Actinobacteriota | Acidimicrobiia | Microtrichales | (s) *Ilumatobacter coccineus* | 1.43 |
| Proteobacteria | Gammaproteobacteria | Granulosicoccales | (g) GCA-1730015 | 1.38 |
| Proteobacteria | Gammaproteobacteria | Pseudomonadales | (g) ASP10-02a | 1.29 |
| Proteobacteria | Alphaproteobacteria | Rhodobacterales | (s) *Roseovarius aestuarii* | 1.24 |
| Actinobacteriota | Acidimicrobiia | Microtrichales | (f) SZUA-35 | 0.91 |
| Proteobacteria | Alphaproteobacteria | Rhodobacterales | (g) HLUCCA08 | 0.91 |
|  |  |  |  |  |
| ***Tank Farm Beach*** | |  |  |  |
| Proteobacteria | Gammaproteobacteria | (Methylococcales | (g) *Cycloclasticus* sp. | 6.50 |
| Proteobacteria | Gammaproteobacteria | Pseudomonadales | (g) GCA-2707785 | 3.32 |
| Acidobacteriota | Blastocatellia | Pyrinomonadales | (g) OLB17 | 3.27 |
| Proteobacteria | Gammaproteobacteria | Pseudomonadales | (g) *Porticoccu*s sp. | 2.18 |
| Proteobacteria | Gammaproteobacteria | Pseudomonadales | (g) HTCC2207 | 1.96 |
| Proteobacteria | Gammaproteobacteria | Pseudomonadales | (g) HTCC2207 | 1.79 |
| Proteobacteria | Gammaproteobacteria | Pseudomonadales | (g) UBA2679 | 1.75 |
| Proteobacteria | Alphaproteobacteria | Rhodobacterales | (s) *Ruegeria* B *marina* | 1.70 |
| Proteobacteria | Gammaproteobacteria | Pseudomonadales | (s) *Marinobacter santoriniensis* | 1.27 |
| Proteobacteria | Gammaproteobacteria | Pseudomonadales | (g) HTCC2207 | 1.22 |
|  |  |  |  |  |
| ***Tupirvik Beach*** |  |  |  |  |
| Proteobacteria | Gammaproteobacteria | Granulosicoccales | (s) *Granulosicoccus antarcticus* | 7.59 |
| Proteobacteria | Gammaproteobacteria | Pseudomonadales | (g) HTCC2207 | 4.13 |
| Proteobacteria | Gammaproteobacteria | Pseudomonadales | (g) ASP10-02a | 3.79 |
| Proteobacteria | Alphaproteobacteria | Rhodobacterales | (g) *Ascidiaceihabitans* sp. | 3.20 |
| Proteobacteria | Gammaproteobacteria | Pseudomonadales | (g) ASP10-02a | 3.04 |
| Proteobacteria | Gammaproteobacteria | Pseudomonadales | (g) HTCC2207 | 2.87 |
| Proteobacteria | Gammaproteobacteria | Pseudomonadales | (g) ASP10-02a | 2.61 |
| Proteobacteria | Alphaproteobacteria | Rhodobacterales | (g) *Planktomarina* sp. | 2.36 |
| Proteobacteria | Gammaproteobacteria | Pseudomonadales | (g) HTCC2207 | 2.11 |
| Bacteroidota | Bacteroidia | Flavobacteriales | (g) SCGC-AAA160-P02 | 1.69 |

**Table S9 (xlsx).** List of completeness pathways present in each MAG from **Fig. S7**. Pathway completeness is indicated by number: absence of the pathway – 0, less than half complete – 1, half complete – 2, more than half complete – 3, and complete pathway – 4.

**Table S10.** List of MAGs and key hydrocarbon degradation genes from **Fig. 7**. Each value represents the number of count per gene in each MAG.

**Table S11.** List of proteins and pathways present in the five selected MAGs from **Fig. 8**.

**
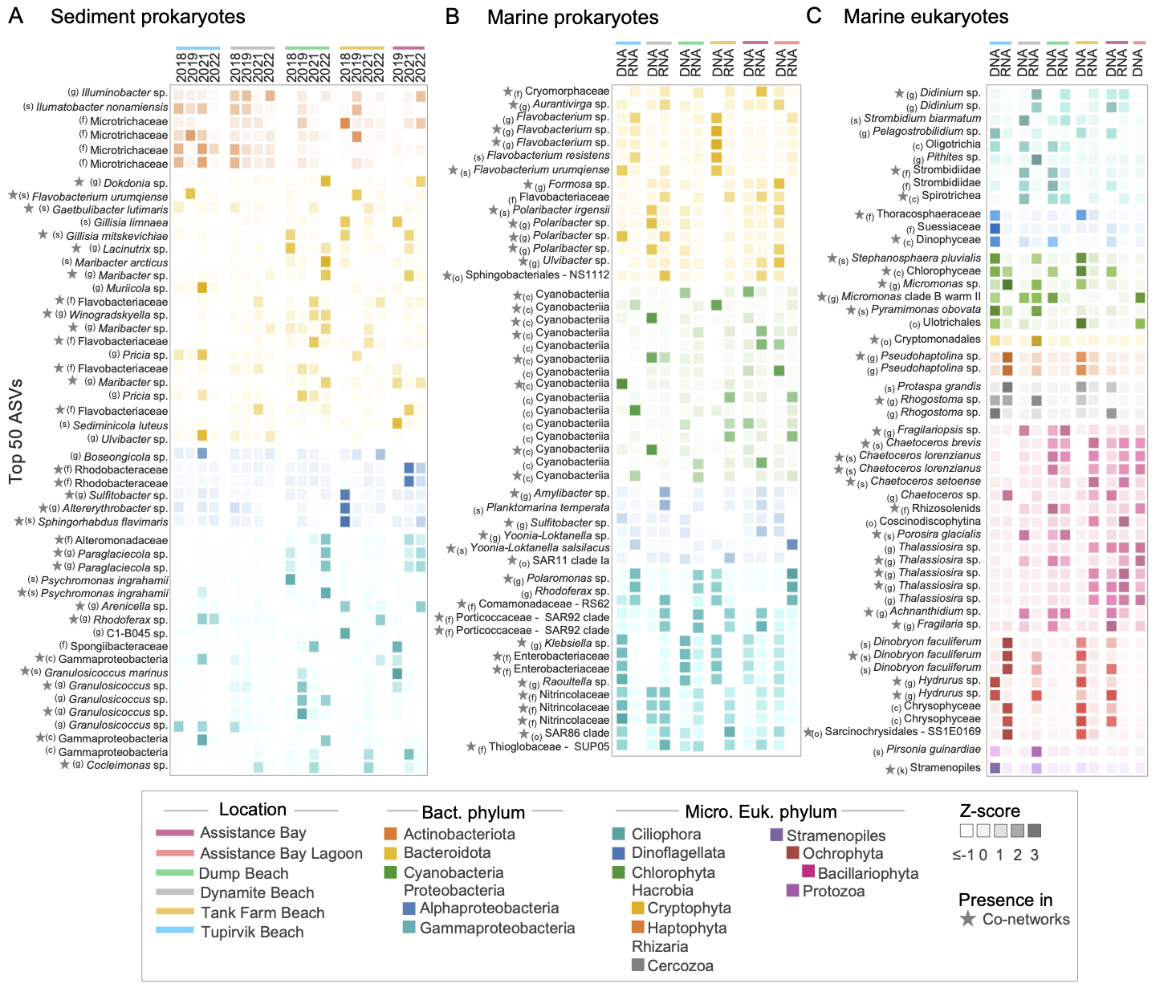
**

**Fig. S1. Heatmap of the top 50 ASVs of coastal microbial community.** Deviation of the relative abundance (z-score) for the **A.** beach sediment prokaryotes in each year at the five beaches; **B.** shoreline marine prokaryotes in July 2022; and **C.** shoreline microbial eukaryotes in July 2022. For each ASV, the Z-score shows the deviation from the mean relative abundance (Z-score = ASV relative abundance – mean relative abundance / standard deviation). Each color represents the phylum level classification of each ASV. Only ASVs of DNA dataset are shown for **A.** sediment samples. ASVs of both DNA and RNA datasets are shown only for **A.** marine bacteria and **C.** marine eukaryotes. Assistance Bay lagoon is located at Assistance Bay (**Table S1**). Each star represents the presence of ASV in both co-occurrence networks of **Figs. 4-5**.


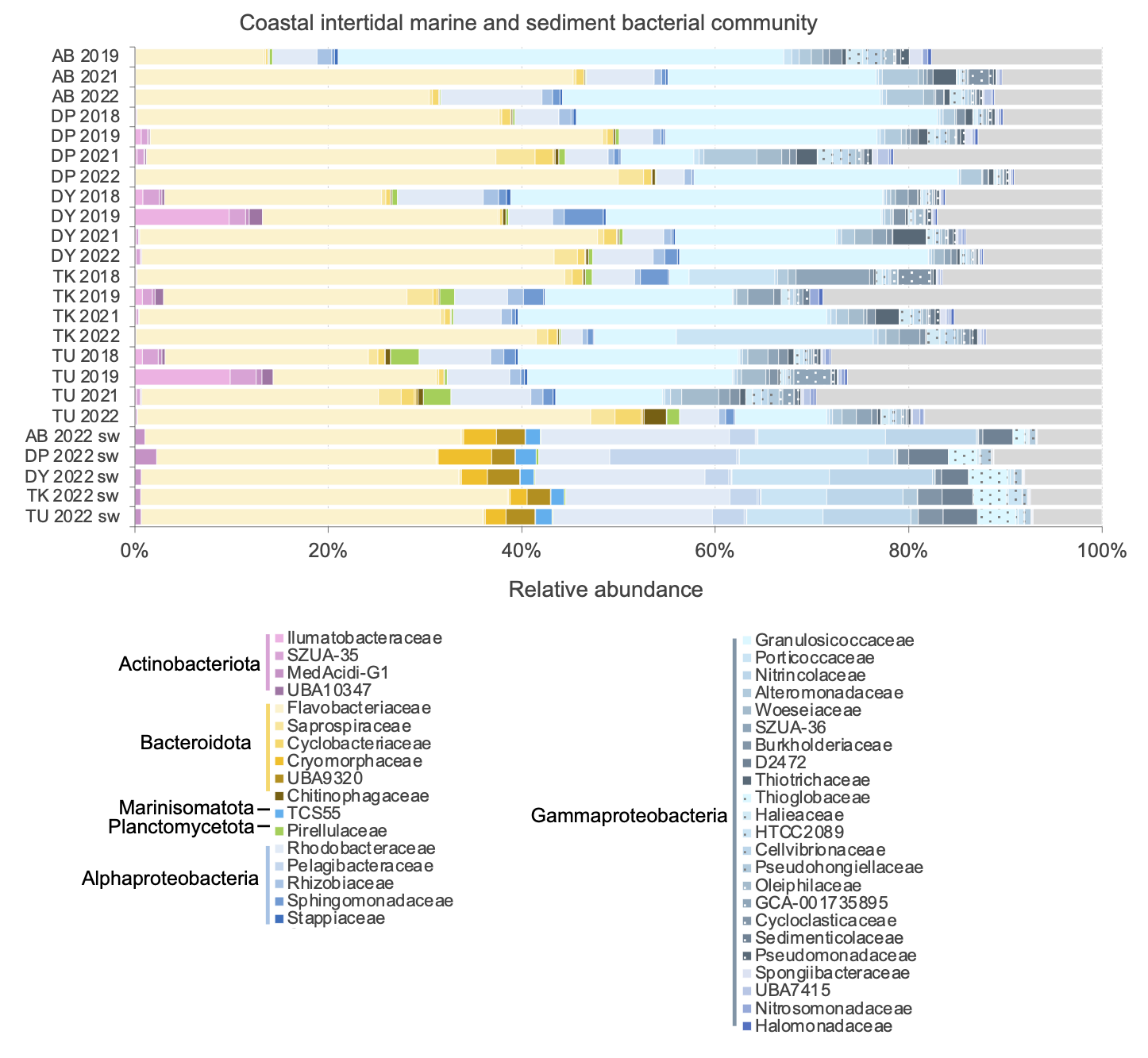


**Fig. S2.** Metagenome read taxonomy of coastal intertidal marine and sediment bacterial communities. Acronyms for sampling beach sites: AB – Assistance Bay, DP – Dump Beach, DY – Dynamite Beach, TK – Tank Farm and TU – Tupirvik Beach. Only seawater samples are indicated by ‘sw’.


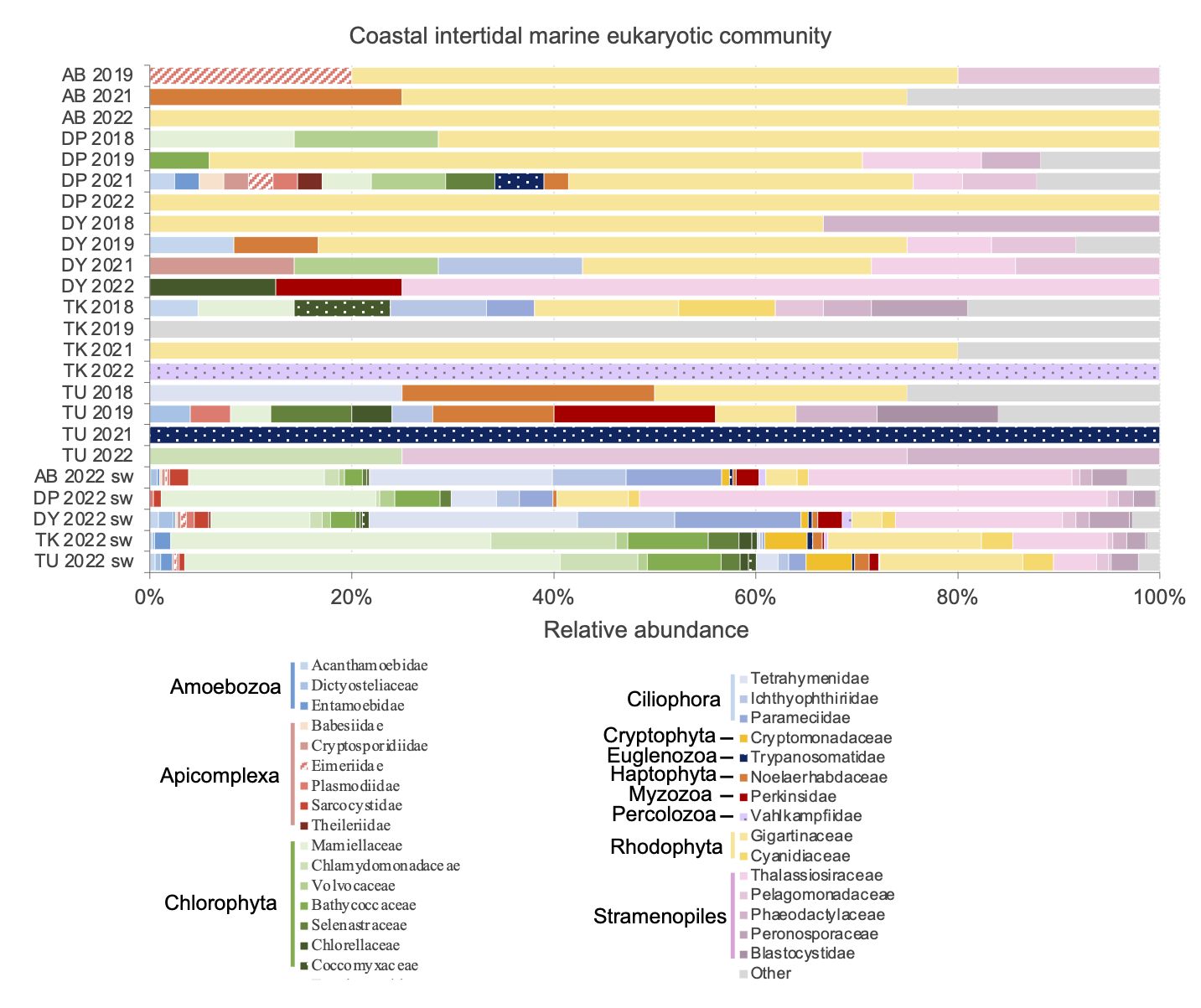


**Fig. S3.** Metagenome read taxonomy of coastal intertidal marine and sediment eukaryotic communities. Acronyms for sampling beach sites: AB – Assistance Bay, DP – Dump Beach, DY – Dynamite Beach, TK – Tank Farm and TU – Tupirvik Beach. Only seawater samples are indicated by ‘sw’.

**
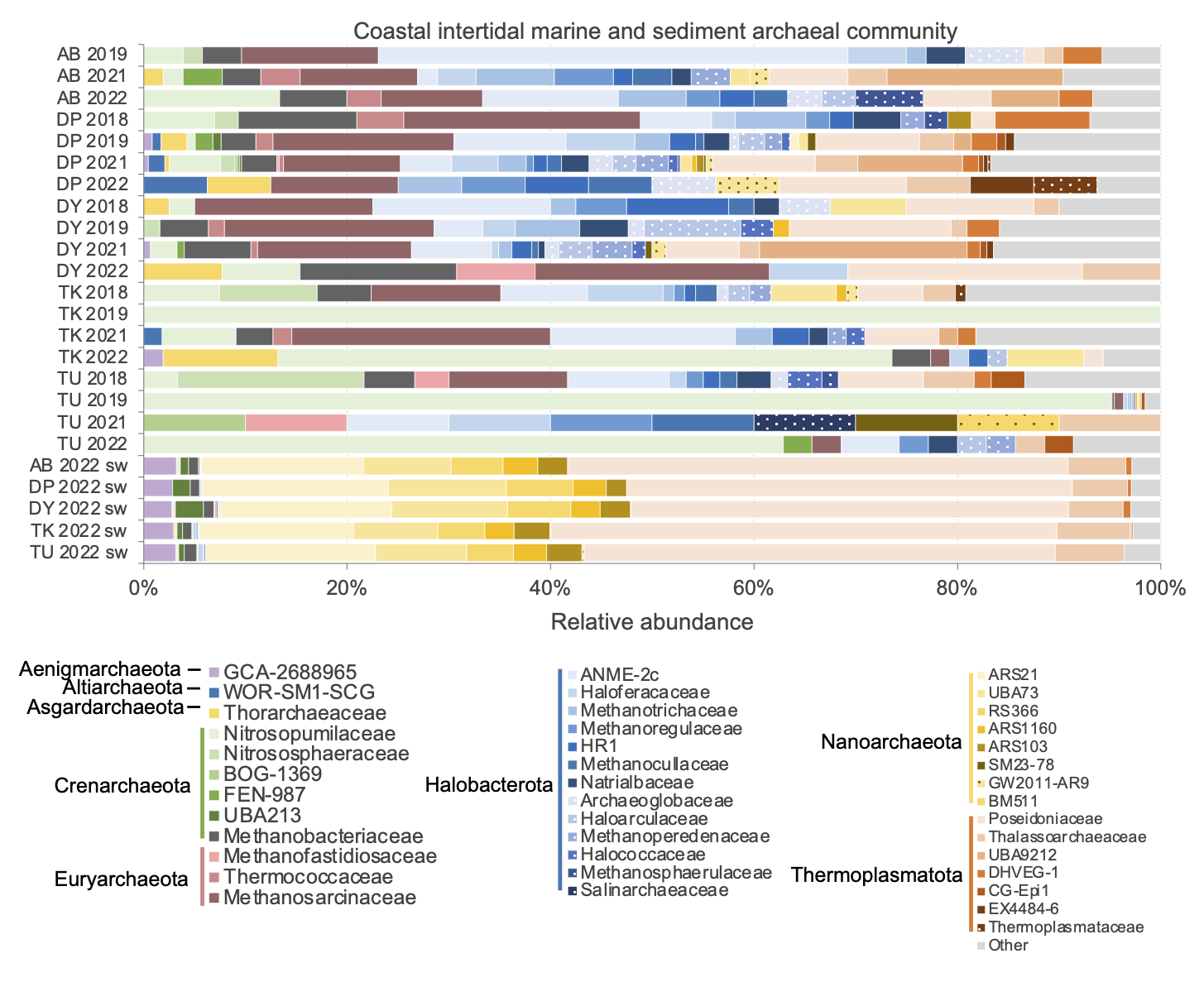
**

**Fig. S4.** Metagenome read taxonomy of coastal intertidal marine and sediment archaeal communities. Acronyms for sampling beach sites: AB – Assistance Bay, DP – Dump Beach, DY – Dynamite Beach, TK – Tank Farm and TU – Tupirvik Beach. Only seawater samples are indicated by ‘sw’.

**
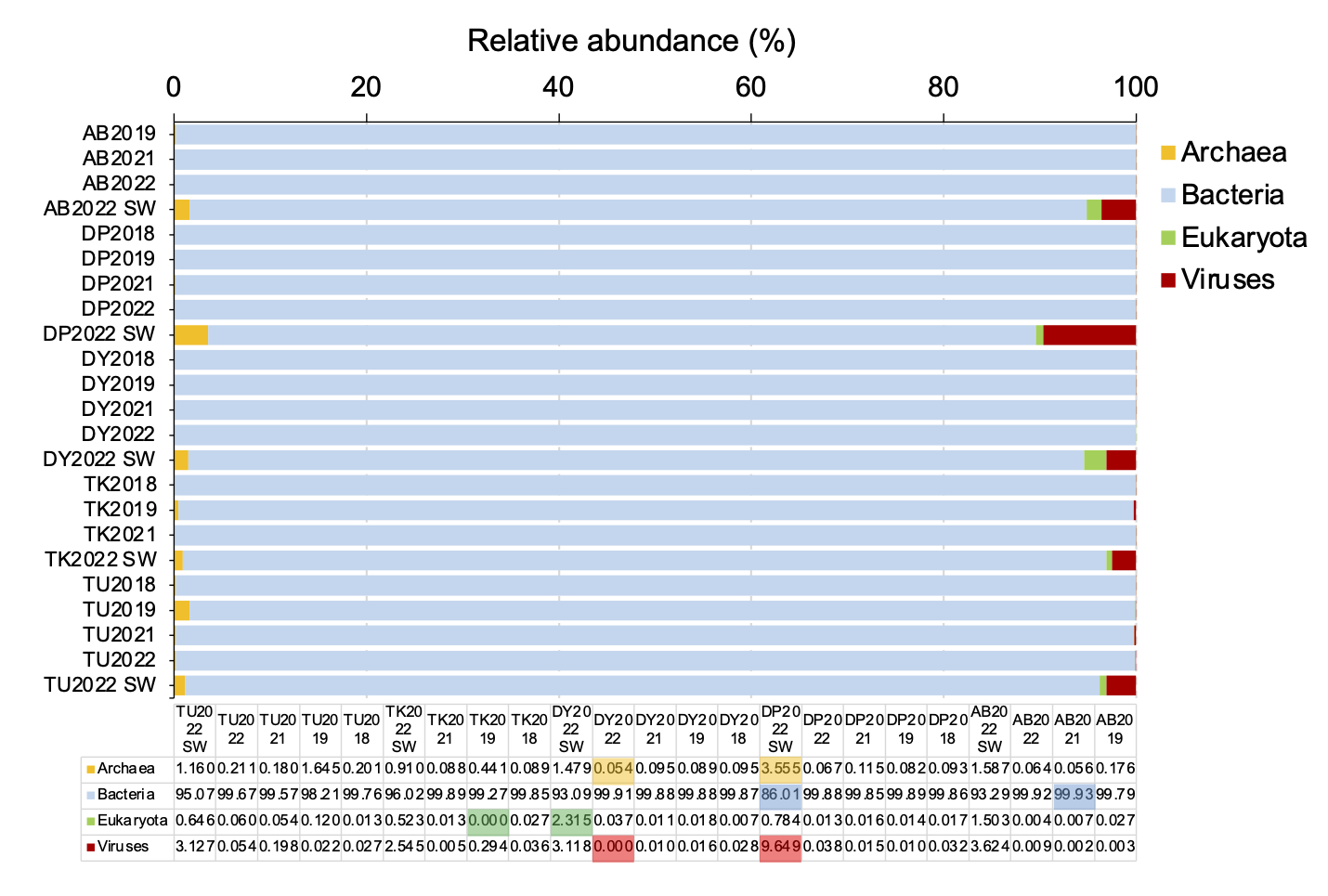
**

**Fig. S5.** Proportion of archaea, bacteria, eukaryota and viruses in nanopore metagenomes of coastal intertidal marine and sediment samples. Acronyms for sampling beach sites: AB – Assistance Bay, DP – Dump Beach, DY – Dynamite Beach, TK – Tank Farm and TU – Tupirvik Beach. Only seawater samples are indicated by ‘sw’.

**
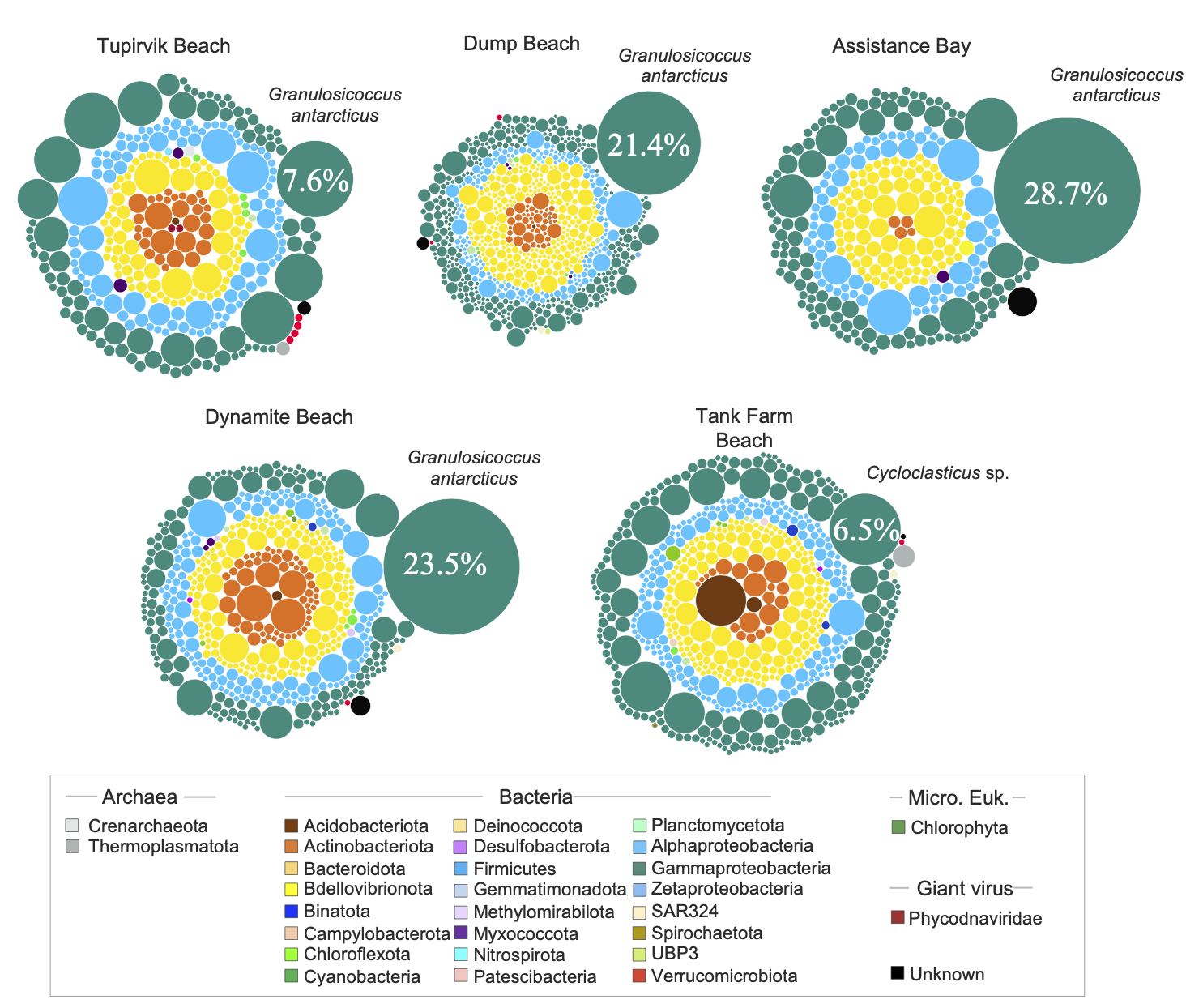
**

**Fig. S6. Hydrocarbon-degraders identified in long-read metagenomes across the coastal Canadian high Arctic.** Circle plots of each beach show the relative abundance in percentage of each genus represented by each dot, within a phylum (color coded). A list of the top10 hydrocarbon-degraders is in **Table S8**.

**
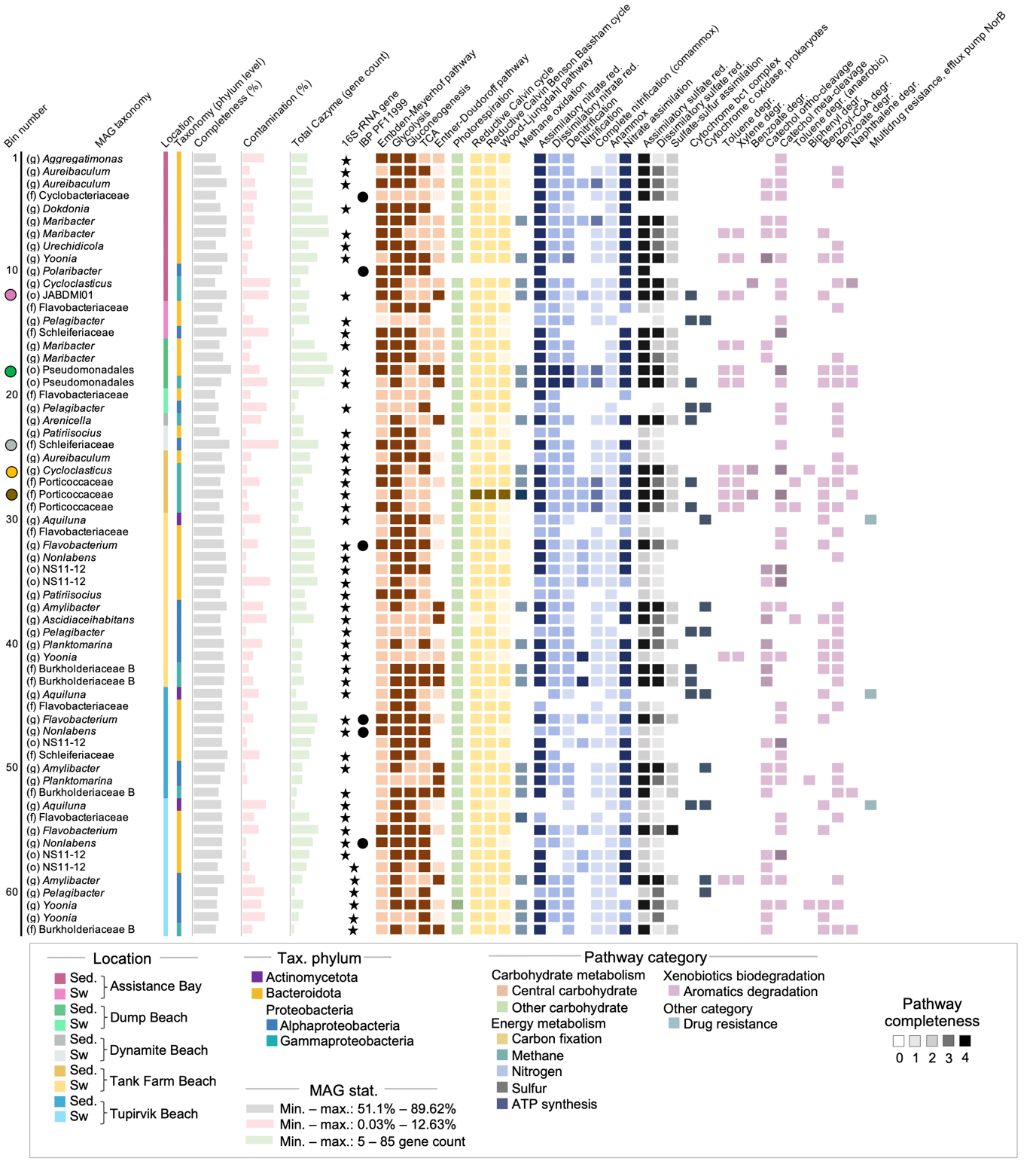
**

**Fig. S7. Metabolic potentials in each metagenome-assembled genome (MAG) affiliated to the lowest taxonomic level.** Pathway completeness is indicated by intensity of the color: absence of the pathway – 0 (white); less than half complete – 1; half complete – 2; more than half complete – 3; and complete pathway – 4 (strongest intensity of color). Each star represents the presence of 16S rRNA gene in each MAG. Black circle indicates the presence of ice-binding protein (IBP), a cold-adapted domain with pfam number 11999. Each MAG is classified to the lowest level assigned by GTDB-tk. The location of sampling site is indicated by colors with “Sed.” indicating sediment samples and “Sw” indicating seawater samples. The five selected MAGs (see **Fig. 8**) are indicated by a colored circle in the bin number column. A corresponding table for each MAG (Supplemental information) is in **Table S3**, and a complete table of completeness of pathways in each MAG is in **Table S8.**

**SUPPLEMENTARY REFERENCES**

Alneberg, J., Bjarnason, B.S., de Bruijn, I., Schirmer, M., Quick, J., Ijaz, U.Z., Loman, N.J., Andersson, A.F., and Quince, C. (2013). CONCOCT: clustering contigs on coverage and composition. arXiv preprint arXiv:13124038.

Bedward, M., and Eppstein, D. (2018). Menzel P. packcircles: circle packing. R package version 03 *3*.

Belzile, C., Brugel, S., Nozais, C., Gratton, Y., and Demers, S. (2008). Variations of the abundance and nucleic acid content of heterotrophic bacteria in Beaufort Shelf waters during winter and spring. J Mar Syst *74*, 946-956.

Bolyen, E., Rideout, J.R., Dillon, M.R., Bokulich, N.A., Abnet, C., Al-Ghalith, G.A., Alexander, H., Alm, E.J., Arumugam, M., and Asnicar, F. (2018). QIIME 2: Reproducible, interactive, scalable, and extensible microbiome data science (PeerJ Preprints).

Brown, B.W., and Russell, K. (1997). Methods correcting for multiple testing: operating characteristics. Statistics in medicine *16*, 2511-2528.

Callahan, B. (2018). Silva taxonomic training data formatted for DADA2 (Silva version 132). Zenodo.

Callahan, B.J., McMurdie, P.J., Rosen, M.J., Han, A.W., Johnson, A.J.A., and Holmes, S.P. (2016). DADA2: High-resolution sample inference from Illumina amplicon data. Nat Methods *13*, 581-583.

Cantarel, B.L., Coutinho, P.M., Rancurel, C., Bernard, T., Lombard, V., and Henrissat, B. (2009). The Carbohydrate-Active EnZymes database (CAZy): an expert resource for glycogenomics. Nucl Acids Res *37*, D233-D238.

Chaumeil, P.-A., Mussig, A.J., Hugenholtz, P., and Parks, D.H. (2020). GTDB-Tk: a toolkit to classify genomes with the Genome Taxonomy Database (Oxford University Press).

Comeau, A.M., Li, W.K., Tremblay, J.-É., Carmack, E.C., and Lovejoy, C. (2011). Arctic Ocean microbial community structure before and after the 2007 record sea ice minimum. PloS One *6*, e27492.

Dong, Q., Mao, K., Duan, D., Zhao, S., Wang, Y., Wang, Q., Huang, D., Li, C., Liu, C., Gong, X.*, et al.* (2018). Genome-wide analyses of genes encoding FK506-binding proteins reveal their involvement in abiotic stress responses in apple. BMC Genom *19*, 707.

Eiler, A., Heinrich, F., and Bertilsson, S. (2012). Coherent dynamics and association networks among lake bacterioplankton taxa. ISME J *6*, 330-342.

Faust, K., and Raes, J. (2016). CoNet app: inference of biological association networks using Cytoscape. F1000Research *5*.

Finn, R.D., Clements, J., and Eddy, S.R. (2011). HMMER web server: interactive sequence similarity searching. Nucleic acids research *39*, W29-W37.

Freyria, N.J., Joli, N., and Lovejoy, C. (2021). A decadal perspective on north water microbial eukaryotes as Arctic Ocean sentinels. Sci Rep *11*, 8413.

Freyria, N.J., Kuo, A., Chovatia, M., Johnson, J., Lipzen, A., Barry, K.W., Grigoriev, I.V., and Lovejoy, C. (2022). Salinity tolerance mechanisms of an Arctic Pelagophyte using comparative transcriptomic and gene expression analysis. Commun Biol *5*, 500.

Guillou, L., Bachar, D., Audic, S., Bass, D., Berney, C., Bittner, L., Boutte, C., Burgaud, G., de Vargas, C., and Decelle, J. (2012). The Protist Ribosomal Reference database (PR2): a catalog of unicellular eukaryote small sub-unit rRNA sequences with curated taxonomy. Nucleic acids research *41*, D597-D604.

Huang, L., Zhang, H., Wu, P., Entwistle, S., Li, X., Yohe, T., Yi, H., Yang, Z., and Yin, Y. (2018). dbCAN-seq: a database of carbohydrate-active enzyme (CAZyme) sequence and annotation. Nucl Acids Res *46*, D516-D521.

Kang, D.D., Li, F., Kirton, E., Thomas, A., Egan, R., An, H., and Wang, Z. (2019). MetaBAT 2: an adaptive binning algorithm for robust and efficient genome reconstruction from metagenome assemblies. PeerJ *7*, e7359.

Khot, V., Zorz, J., Gittins, D.A., Chakraborty, A., Bell, E., Bautista, M.A., Paquette, A.J., Hawley, A.K., Novotnik, B., and Hubert, C.R. (2022). CANT-HYD: a curated database of phylogeny-derived hidden Markov models for annotation of marker genes involved in hydrocarbon degradation. Front microbiol *12*, 764058.

Kolmogorov, M., Bickhart, D.M., Behsaz, B., Gurevich, A., Rayko, M., Shin, S.B., Kuhn, K., Yuan, J., Polevikov, E., and Smith, T.P. (2020). metaFlye: scalable long-read metagenome assembly using repeat graphs. Nat Methods *17*, 1103-1110.

Legendre, P., and Legendre, L. (1998). 1998. Numerical ecology. Second English edition Elsevier, Amsterdam.

Magnuson, E., Altshuler, I., Freyria, N.J., Leveille, R.J., and Whyte, L.G. (2023). Sulfur-cycling chemolithoautotrophic microbial community dominates a cold, anoxic, hypersaline Arctic spring. Microbiome *11*, 203.

Marie, D., Simon, N., Guillou, L., Partensky, F., and Vaulot, D. (2000). DNA/RNA analysis of phytoplankton by flow cytometry. Curr Protoc Cytom *11*, 11-12.

Oksanen, J. (2013). Vegan: ecological diversity. *R project 368*, 1-11.

Olm, M.R., Brown, C.T., Brooks, B., and Banfield, J.F. (2017). dRep: a tool for fast and accurate genomic comparisons that enables improved genome recovery from metagenomes through de-replication. ISME J *11*, 2864-2868.

Parada, A.E., Needham, D.M., and Fuhrman, J.A. (2016). Every base matters: assessing small subunit rRNA primers for marine microbiomes with mock communities, time series and global field samples. Environmental microbiology *18*, 1403-1414.

Parks, D.H., Imelfort, M., Skennerton, C.T., Hugenholtz, P., and Tyson, G.W. (2015). CheckM: assessing the quality of microbial genomes recovered from isolates, single cells, and metagenomes. Genome Res *25*, 1043-1055.

Quast, C., Pruesse, E., Yilmaz, P., Gerken, J., Schweer, T., Yarza, P., Peplies, J., and Glöckner, F.O. (2012). The SILVA ribosomal RNA gene database project: improved data processing and web-based tools. Nucl Acids Res *41*, D590-D596.

Schlitzer, R. (2015). Data analysis and visualization with Ocean Data View. CMOS Bulletin SCMO *43*, 9-13.

Shannon, P., Markiel, A., Ozier, O., Baliga, N.S., Wang, J.T., Ramage, D., Amin, N., Schwikowski, B., and Ideker, T. (2003). Cytoscape: a software environment for integrated models of biomolecular interaction networks. Genome Res *13*, 2498-2504.

Team, R.C., Team, M.R.C., Suggests, M., and Matrix, S. (2018). Package stats. In The R Stats Package.

Thissen, D., Steinberg, L., and Kuang, D. (2002). Quick and easy implementation of the Benjamini-Hochberg procedure for controlling the false positive rate in multiple comparisons. Journal of educational and behavioral statistics *27*, 77-83.

Wei, T., Simko, V., Levy, M., Xie, Y., Jin, Y., and Zemla, J. (2017). Package ‘corrplot’. Statistician *56*, e24.

Wickham, H. (2011). ggplot2. Wiley interdisciplinary reviews: computational statistics *3*, 180-185.

Wickham, H., François, R., Henry, L., Müller, K., and Wickham, M.H. (2019). Package ‘dplyr’. A Grammar of Data Manipulation R package version *8*.

Wright, K., and Wright, M.K. (2018). Package ‘corrgram’. Plot a Correlogram.

Wu, Y.-W., Tang, Y.-H., Tringe, S.G., Simmons, B.A., and Singer, S.W. (2014). MaxBin: an automated binning method to recover individual genomes from metagenomes using an expectation-maximization algorithm. Microbiome *2*, 1-18.
